# Adaptation of gene loci to heterochromatin in the course of *Drosophila* evolution is associated with insulator proteins

**DOI:** 10.1038/s41598-020-68879-2

**Published:** 2020-07-17

**Authors:** Sergei Yu. Funikov, Alexander P. Rezvykh, Dina A. Kulikova, Elena S. Zelentsova, Lyudmila A. Protsenko, Lyubov N. Chuvakova, Venera I. Tyukmaeva, Irina R. Arkhipova, Michael B. Evgen’ev

**Affiliations:** 1 Engelhardt Institute of Molecular Biology of Russian Academy of Sciences, Moscow, 119991 Russia; 2 Moscow Institute of Physics and Technology, Dolgoprudny, Moscow Region Russia; 3 Koltzov Institute of Developmental Biology of Russian Academy of Sciences, Moscow, Russia; 4 Department of Biological and Environmental Science, University of Jyväskylä, 40014 Jyväskylä, Finland; 5 Josephine Bay Paul Center for Comparative Molecular Biology and Evolution, Marine Biological Laboratory, Woods Hole, MA USA

**Keywords:** Evolutionary genetics, Molecular evolution, Evolution, Molecular biology, Chromatin, Transcription

## Abstract

Pericentromeric heterochromatin is generally composed of repetitive DNA forming a transcriptionally repressive environment. Dozens of genes were embedded into pericentromeric heterochromatin during evolution of *Drosophilidae* lineage while retaining activity. However, factors that contribute to insusceptibility of gene loci to transcriptional silencing remain unknown. Here, we find that the promoter region of genes that can be embedded in both euchromatin and heterochromatin exhibits a conserved structure throughout the *Drosophila* phylogeny and carries motifs for binding of certain chromatin remodeling factors, including insulator proteins. Using ChIP-seq data, we demonstrate that evolutionary gene relocation between euchromatin and pericentric heterochromatin occurred with preservation of sites of insulation of BEAF-32 in evolutionarily distant species, i.e. *D. melanogaster* and *D. virilis*. Moreover, promoters of virtually all protein-coding genes located in heterochromatin in *D. melanogaster* are enriched with insulator proteins BEAF-32, GAF and dCTCF. Applying RNA-seq of a BEAF-32 mutant, we show that the impairment of BEAF-32 function has a complex effect on gene expression in *D. melanogaster,* affecting even those genes that lack BEAF-32 association in their promoters. We propose that conserved intrinsic properties of genes, such as sites of insulation near the promoter regions, may contribute to adaptation of genes to the heterochromatic environment and, hence, facilitate the evolutionary relocation of genes loci between euchromatin and heterochromatin.

## Introduction

Eukaryotic genomes are packaged in chromatin consisting of DNA, RNA and associated proteins. Typically, chromatin can be divided into two basic forms, euchromatin and heterochromatin^1^. These types of chromatin are distinguished by several distinctive properties, including DNA sequence composition, specific histone modifications and binding proteins, nuclear and chromosomal localization, rate of DNA replication and frequency of meiotic recombination^1,2^. One of the major subtypes of heterochromatin in *Drosophila* is marked by heterochromatin protein 1 (HP1a) and di- or trimethylated H3K9^3,4^. This subtype of heterochromatin covers large genomic segments primarily around centromeres and, in association with the protein POF (painting of fourth), the entire dot chromosome 4 in *D. melanogaster*^3–5^. Pericentric heterochromatin is mainly composed of repetitive sequences, including remnants of various transposable elements (TEs) and satellite DNAs^6^. A distinctive feature of heterochromatin is the ability to silence euchromatic genes placed within heterochromatic environment due to chromosomal inversions or transposition events, a phenomenon called position effect variegation (PEV)^7–12^. It is generally believed that transcriptional silencing of euchromatic genes in PEV is mediated by spreading of heterochromatin-associated marks HP1a and H3K9me3 across the gene loci transferred to heterochromatin^8,10^.

Despite the repressive environment, dozens of essential genes were identified in the pericentric heterochromatin of *D. melanogaster*^13–16^. Interestingly, genes embedded in pericentric heterochromatin in *D. melanogaster* may occupy distinct genomic regions, euchromatic and heterochromatic, in other *Drosophila* species^17^. For instance, two adjacent genes *RpL15* and *Dbp80* located in the pericentric region of chromosome 3L in *D. melanogaster* reside in a euchromatic region in *D. pseudoobscura*^18^. A similar pattern was demonstrated for genes *light* and *Yeti* located in pericentric regions in *D. melanogaster,* while in *D. virilis* they are found within euchromatin on the same chromosomal elements^19,20^. Recently, it was shown that most of the pericentric genes found at both arms of chromosome 2 of *D. melanogaster* are located in euchromatic region in the *D. virilis* genome^21^. However, although relocation of genes between euchromatin and heterochromatin during genome evolution is not unusual in the *Drosophilidae* lineage, the insusceptibility of heterochromatic genes to transcriptional silencing remains paradoxical and unexplained. It is still not clear whether pericentric gene loci have undergone adaptation to heterochromatic environment or originally had some intrinsic properties permitting local adaptation.

Chromatin insulator elements and associated proteins were originally defined by their ability to protect transgenes from PEV^22–25^. Numerous studies demonstrated that insulator proteins are responsible for a vast number of genomic functions, including stimulation of gene transcription, enhancer-blocking and barrier insulation partitioning of eukaryotic genomes into independently regulated domains^26–28^. Hence, one may hypothesize that gene loci capable of adaptation to heterochromatin probably share specific sites of insulation that ensure their expression in the repressive environment.

To address this issue, we initially investigated the molecular evolution of *Myb* and *Ranbp16* genes which were relocated between euchromatic and heterochromatic environment in the *Drosophilidae* lineage. Further, we examined the regulatory factors that contribute to normal functioning of genes relocated into heterochromatic locations in distant *Drosophila* species, e.g. *D. melanogaster* and *D. virilis*. *Myb* is an essential gene encoding a transcription factor involved in transition from G2 to M phase of the cell cycle^29,30^. *Ranbp16* encodes a RanGTP-binding protein belonging to the importin-β superfamily and mediates translocation of proteins into the nucleus. Both genes are located in euchromatic region of the *D. melanogaster* X-chromosome, while in other studied *Drosophila* species belonging to Sophophora and Drosophila subgenus they are found in genomic regions with a high density of repetitive DNA elements, suggesting their localization in heterochromatin. We found that regardless of the euchromatic or heterochromatic surroundings, the promoter region of *Myb* displays a high degree of sequence homology among *Drosophila* species studied so far. The conserved motifs in the promoter sequence of *Myb* serve as a binding site for the chromatin insulator protein BEAF-32 (Boundary element associated factor of 32 kDa) and transcriptional factor Dref (The DNA replication-related element (DRE) binding factor). Using ChIP-seq data, we demonstrate that the insulator protein BEAF-32 occupies promoters of the same genes which are located in contrasting chromatin types in *D. melanogaster* and *D. virilis,* denoting the boundary of the nucleosome-free region available for RNA polymerase II recruitment and the surrounding heterochromatin. Moreover, our analysis revealed that promoters of practically all protein-coding genes located in heterochromatin in *D. melanogaster* are enriched with insulator proteins BEAF-32, GAF (GAGA factor) and dCTCF (*Drosophila* homolog of CCCTC-binding factor). Exploring available RNA-seq data of mutant BEAF-32 function in Drosophila cells, we show that deficiency of BEAF-32 has a complex effect on expression of most genes in the genome, including heterochromatic ones. Overall, we propose that insulator proteins, in particular BEAF-32, are linked to expression of heterochromatic genes and may facilitate their normal function after evolutionary relocation into transcriptionally repressive genomic environment.

## Results

### Evolutionary relocation of *Myb* and *Ranbp16* genes between euchromatin and heterochromatin in *Drosophila* phylogeny

**Figure 1:**
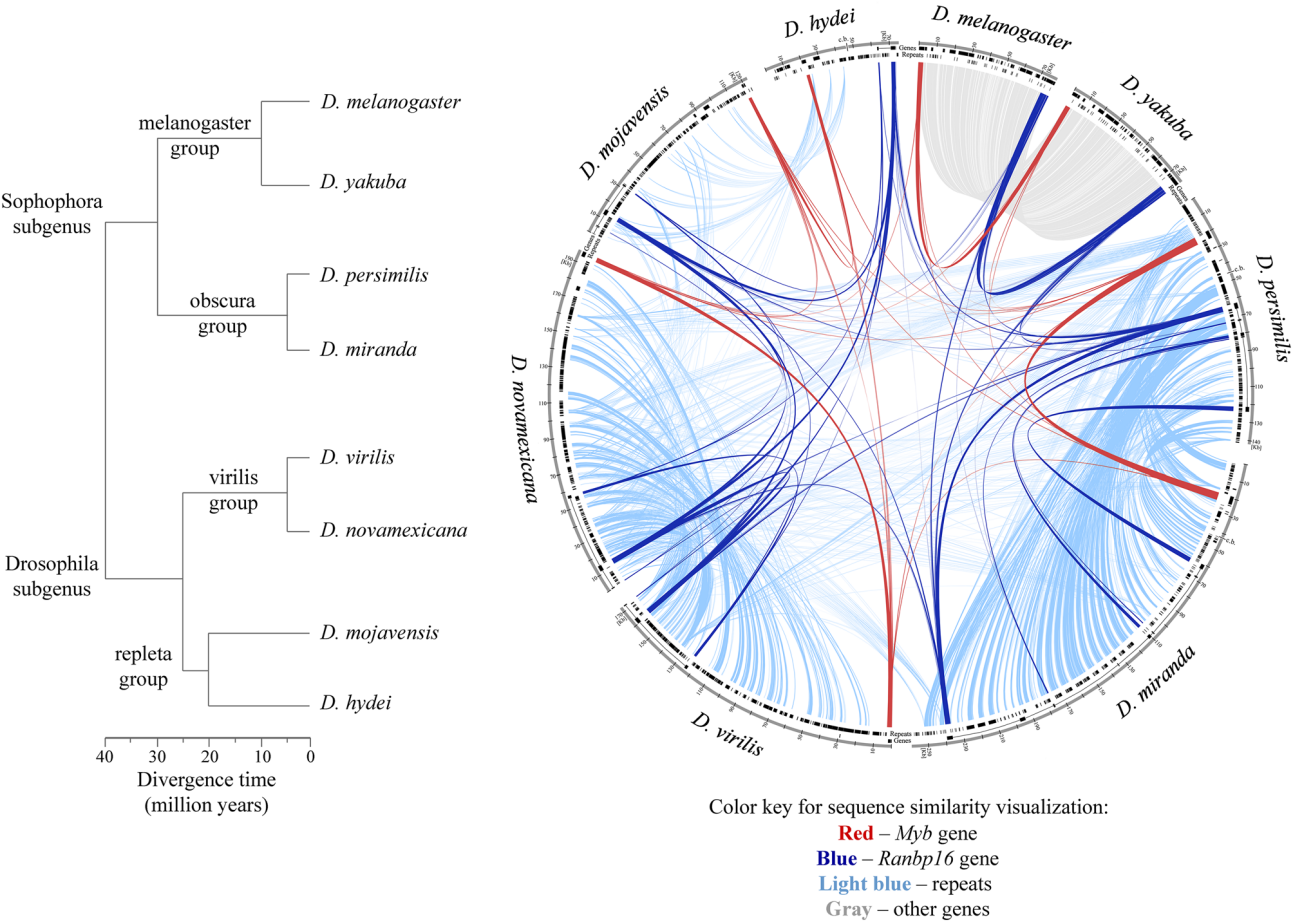
Similarities and differences of genomic regions comprising *Myb* and *Ranbp16* in *Drosophila* species. On the left—the phylogenetic tree indicating the relationships among studied species with estimated times of divergence according to Clark et al.^31^, Gao et al.^32^ for the obscura group, O’Grady et al.^35^ for the virilis group and Gibbs et al.^33^ for the repleta group. On the right—circular plot demonstrating similarity of *Myb* (red nodes) and *Ranbp16* (blue nodes) and diversity of intergenic regions between these genes consisting of repeats (light blue nodes) and protein-coding genes (gray nodes) among the species of the Sophophora and Drosophila subgenera. Tracks of the plot indicate the region of comparison, coordinates of genes including *Myb* and *Ranbp16,* as well as the content of annotated repeats in the plotted region. Flanking regions of *Myb* and *Ranbp16* (20 Kb upstream and downstream from the gene location) were used instead of intergenic regions, due to the long distance between these genes in *D. miranda* (> 17 Mb) and low scaffold contiguity around these genes for *D. persimilis* and *D. hydei*. These contigs borders are shown by lines (signed c.b., contig borders).

In order to determine whether *Myb* and *Ranbp16* gene locations have been rearranged on the evolutionary timescale, we first mapped these genes onto genomic scaffolds of *Drosophila* species separated by evolutionary distances from 5 to 40 million years^31–35^. These include species of the melanogaster group (*D. melanogaster* and *D. yakuba*) and the obscura group (*D. persimilis* and *D. miranda*), with both groups belonging to the Sophophora subgenus, along with the virilis group (*D. virilis* and *D. novamexicana*) and the repleta group (*D. mojavensis* and *D. hydei*) that belong to the Drosophila subgenus (Table S1). Next, we performed comparative analysis of *Myb* and *Ranbp16* genes, as well as the intergenic regions between these genes. As indicated in Fig. 1, the coding sequences of *Myb* and *Ranbp16* genes are highly homologous between the species studied (Fig. 1). However, the regions between *Myb* and *Ranbp16* genes differ significantly, exhibiting sequence similarity only within the related groups. For instance, while *Myb* and *Ranbp16* genes of *D. melanogaster* and *D. yakuba* are embedded within a large gene cluster, their orthologues in other *Drosophila* species reside in the genomic regions mostly occupied by repetitive sequences, including the introns of *Ranbp16* gene (Fig. 1). Thus, *Myb* and *Ranbp16* genes in the species belonging to the melanogaster group are located in euchromatin, in the region containing a large gene cluster. On the contrary, the localization of the studied genes in the environment packed with repetitive elements is typical for most other *Drosophila* species studied here including the virilis, obscura and repleta groups (Fig. 1).

**Figure 2:**
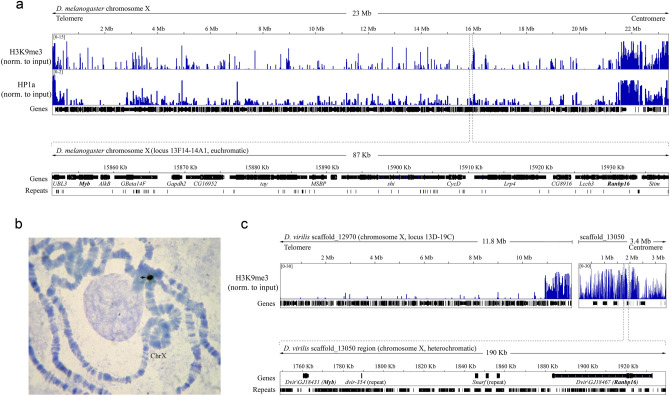
Analysis of genomic regions comprising *Myb* and *Ranbp16* genes in *D. melanogaster* and *D. virilis*. (**a**) Genomic map of whole assembled chromosome X of *D. melanogaster* with mapped ChIP-seq reads of heterochromatic markers H3K9me3 and HP1a and the region depicting *Myb* and *Ranbp16* gene location (marked with bold font). (**b**) DNA in situ hybridization of *Myb* gene probe to polytene chromosomes of *D. virilis*. Black arrow indicates the hybridization signal at the base of chromosome X of *D. virilis*. (**c**) Genomic map of scaffold_13050 of *D. virilis* and the proximal scaffold_12970 which is attributed to chromosome X of *D. virilis* according to Schaeffer et al.^36^ with mapped ChIP-seq reads of H3K9me3 and the region containing *Myb* and *Ranbp16* genes (marked with bold font) on the larger scale. ChIP-seq reads are shown in RPMs (reads per million) normalized to input samples.

Next, we studied in more detail the genomic loci containing *Myb* and *Ranbp16* genes, focusing on two evolutionarily distant species, *D. melanogaster* and *D. virilis,* separated by 40 million years of evolution^31^. The single copies of *Myb* and *Ranbp16* genes map to the chromosome X of *D. melanogaster* at the cytogenetic areas 13F14 and 14A1 of salivary gland polytene chromosomes, respectively (Fig. 2a). These regions are significantly less enriched with heterochromatic marks H3K9me3 and HP1a than telomeric and pericentric regions of the chromosome and located at the distance more than 6 Mb from the heterochromatin–euchromatin border delineated in Riddle et al.^4^. Hence, these regions represent typical euchromatin in this species. As mentioned above, *Myb* and *Ranbp16* genes in *D. melanogaster* are located at a distance ~ 80 Kb from each other within a large protein-coding gene cluster, which includes only a few repetitive sequences (Fig. 2a).

To confirm that *Myb* and *Ranbp16* reside in a heterochromatic region in *D. virilis,* we performed DNA in situ hybridization on polytene chromosomes of this species using the unique sequence of *Myb* gene as a probe. In situ hybridization indicates that *Myb* is located at the base of chromosome X near the chromocenter in *D. virilis* (Fig. 2b). Mapping analysis of *Myb* and *Ranbp16* genes on the genomic scaffolds of *D. virilis* reveals that both genes reside in one scaffold_13050 at a distance ~ 120 Kb from each other (Fig. 2c). In contrast to *D. melanogaster*, the region between these genes in *D. virilis* does not contain any other protein-coding genes and consists of remnants of TEs and other repeats diverged to varying degrees (Fig. S1). We used annotation of known genomic scaffolds of *D. virilis* made by Schaeffer et al.^36^ to assign the proximal scaffold of *D. virilis* genome r1.06 to the centromeric region of chromosome X. According to specific marker genes (*para* and *tRNA:S7* located at the cytogenetic locus 19B), we retrieved scaffold_12970 and extended it with scaffold_13050 containing *Myb* and *Ranbp16* genes, keeping some space unassembled between these scaffolds (Fig. 2c). Enrichment profile of H3K9me3 clearly indicates that the putative euchromatin-heterochromatin border lies in the proximal 1 Mb of scaffold_12970 (Fig. 2c). The whole scaffold_13050, including the region where *Myb* and *Ranbp16* are localized, is heavily occupied by the H3K9me3 mark in comparison with the most contiguous fragment of scaffold_12970 (Fig. 2c).

To evaluate the possible impact of heterochromatic location on molecular evolution of *Myb* and *Ranbp16*, we examined whether their coding sequences underwent negative (purifying) or positive selection during Drosophila evolution. To this end, we first estimated the number of base substitutions per site in their coding sequences (Fig. S2a,b), and then calculated the ratio of non-synonymous to synonymous substitutions (dN/dS) (Fig. S2c,d) using eight aforementioned representatives of the Sophophora and Drosophila subgenera, as well as the sequences from *Anopheles gambiae* as an outgroup (Fig. S2). The results suggest that both *Myb* and *Ranbp16* genes are under purifying (negative) selection (dN/dS < 0.2 in all pairs of comparison) when present either in heterochromatin or euchromatin.

### Insulator protein BEAF-32 is enriched near the promoters of *Myb* and *Ranbp16* genes in both *D. melanogaster* and *D. virilis*

**Figure 3:**
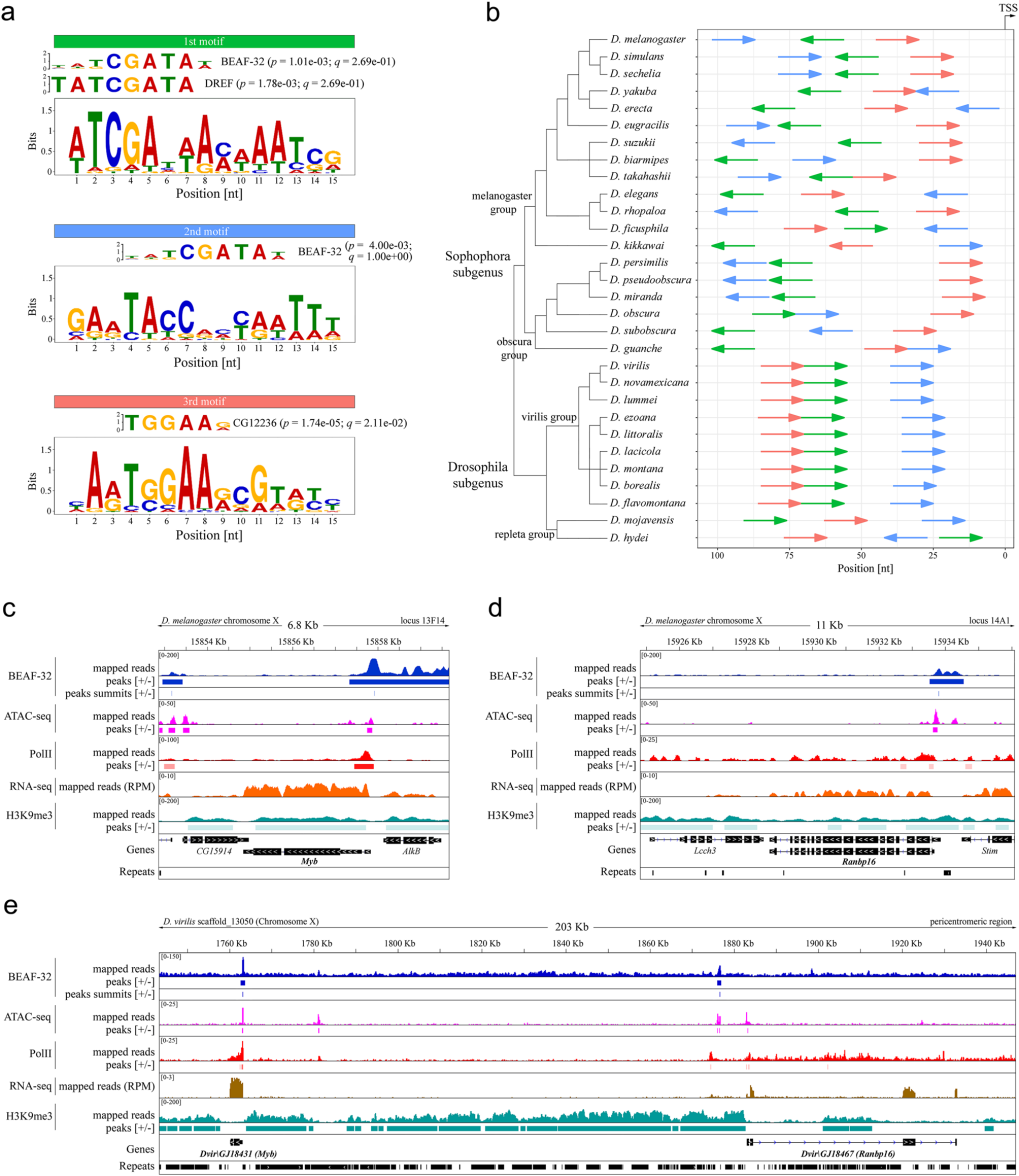
Promoter analysis of *Myb* and *Ranbp16* in *D. melanogaster* and *D. virilis*. (**a**) Sequence logos of the common motifs among the studied *Drosophila* species with matches between motifs and binding sites of known transcription factors. Logos for binding sites of the factors are shown with the corresponding *p*- and *q*-values. (**b**) Distribution of common motifs and their orientation (shown by colored arrows) in the promoter of *Myb*. Prior to the analysis, orientation of all sequences was adjusted so that the transcription start site (TSS) would be on the right. The colors of motifs in A and B correspond to each other. Bootstrap consensus phylogenetic tree is given according to Clark et al.^31^, Gao et al.^32^, O’Grady et al.^35^ and Jezovit et al.^34^. (**c**) and (**d**) Enrichment of ChiP-seq reads within *Myb* and *Ranbp16* genic loci in *D. melanogaster*, respectively. (**e**) The enrichment profile within the *Myb* and *Ranpb16* genic loci and the intergenic regions between these genes in *D. virilis*. Mapped ChIP-seq reads without normalization (mapped reads), calculated areas of enrichment relative to the input data (peaks; P < 0.05) are shown for BEAF-32, Pol II, H3K9me3 and ATAC-seq data. Summits of the enriched reads are shown for BEAF-32 (peaks summits). RNA-seq reads were normalized to the sequence depth (RPM, reads per million).

To reveal specific properties of gene loci allowing the essential *Myb* and *Ranbp16* genes to be actively transcribed in both euchromatin and heterochromatin, we studied the promoter region of *Myb* by searching for common motifs in *Drosophila* species. For this purpose, we expanded the list of analyzed species to 19 representatives of the Sophophora and 11 representatives of the Drosophila subgenera. As mentioned above, due to the absence of gene annotation for a range of studied species (*D. miranda*, *D. guanche*, *D. subobscura* and all virilis group species with the exception of *D. virilis*) the putative TSS was set as the first mapped nucleotide of 5′UTR of *Myb* gene of related species. Using the MEME suite^37^, we were able to identify three motifs that are present in the promoter of *Myb* gene in all analyzed species of *Drosophila* (Fig. 3a, b). Search through OnTheFly^38^ and REDfly v5.6^39,40^ databases of known transcription factors and their binding sites indicates that one highest-scoring motif contains a potential binding site for insulator protein BEAF-32 and transcriptional factor Dref (1st motif, Fig. 3a). The two other motifs show limited similarity to the additional BEAF-32 motif and binding site of undescribed C2H2-type zinc finger transcription factor (Fig. 3a).

To confirm that the insulator protein BEAF-32 binds to the promoter region of *Myb* gene, we used ChIP-seq data to profile BEAF-32 occupancy in the gene loci containing *Myb* and *Ranbp16* genes in *D. melanogaster* and *D. virilis*. Additionally, we applied ChIP-seq to profile RNA polymerase II (Pol II) distribution in the promoters of *Myb* and *Ranbp16* genes as well as ATAC-seq data to correlate BEAF-32 and Pol II enrichment with the nucleosome-free conformation of chromatin in the promoters of these genes, indicative of intensive transcription. Mapping of RNA-seq and H3K9me3 ChIP-seq data also provides valuable information regarding expression levels of these genes and the heterochromatic profile of the analyzed gene loci, respectively.

As seen in Fig. 3c, in *D. melanogaster* the locus containing *Myb* gene and the adjacent *AlkB* gene is highly enriched with BEAF-32, with the peak summit of mapped BEAF-32 reads in the promoter of the *Myb* gene (Fig. 3c). The enrichment of Pol II and mapped ATAC-seq reads reside downstream from the peak of BEAF-32 binding of the promoter region of *Myb,* probably indicating that BEAF-32 binds to DNA at the boundary of the *Myb* gene locus (Fig. 3c). Likewise, the promoter of *Ranbp16* gene of *D. melanogaster* is enriched with BEAF-32, located slightly upstream from the Pol II binding site and open chromatin region (Fig. 3d).

A similar pattern of BEAF-32 occupancy in the promoters of heterochromatic *Myb* and *Ranbp16* genes is observed in *D. virilis* (Fig. 3e). However, the enrichment profile of BEAF-32 upstream of the TSS of *Ranbp16* gene in *D. virilis* and *D. melanogaster* has one difference which is worth mentioning. In contrast to *D. melanogaster*, where BEAF-32 is enriched in close proximity to the TSS of *Ranbp16*, the binding of BEAF-32 to DNA in *D. virilis* is observed only at a distance of ~ 6.5 Kb from the TSS of *Ranbp16* (Fig. 3e). Notably, within this range, three copies of Jockey transposon are located. According to the data obtained by CAGE-seq (Cap Analysis Gene Expression), *Ranbp16* gene in *D. melanogaster* has two TSS located at a distance of ~ 200 bp from each other, giving rise to slightly distinct transcripts in terms of the length of their 5′UTR (Fig. S3). Given that BEAF-32 is enriched within the upstream promoter of *Ranbp16* in *D. melanogaster*, we assumed that in *D. virilis* the distance to the upstream promoter has been extended due to the transposon insertions that may be spliced in the course of transcription. However, we failed to observe more than a single TSS by 5′RACE analysis at the larval and imago stages as well as in the gonads of *D. virilis,* suggesting either loss of the second promoter or its extremely low efficacy. Interestingly, H3K9me3 is present within the gene bodies of *Myb* and *Ranbp16* genes including exons in *D. melanogaster* (Fig. 3c,d). In turn, in *D. virilis* H3K9me3 is lacking in *Myb* and present only in introns of *Ranbp16* that are enriched with repetitive elements (Fig. 3e).

These results indicate that the insulator protein BEAF-32 is enriched in the vicinity of the promoters of *Myb* gene embedded in distinct chromatin structures (euchromatic vs heterochromatic) in *D. melanogaster* and *D. virilis*. Notably, while binding of BEAF-32 is observed in the promoter of *Ranbp16* gene in *D. melanogaster*, in the case of *D. virilis* the active binding site of BEAF-32 has moved upstream of the proximal observed TSS of *Ranbp16* gene apparently due to transposon insertions.

### Binding of BEAF-32 near the transcription start sites is preserved in the course of evolutionary relocation of gene loci between euchromatin and heterochromatin

Considering the above results, two important questions may be asked. First, do the BEAF-32 binding sites in the vicinity of promoter regions represent a peculiar property of *Myb* and *Ranbp16* genes, or are they a common feature of heterochromatic genes? Second, do the BEAF-32 binding sites found in these promoters emerge in the course of adaptive evolution of genes transposed to heterochromatin? Alternatively, they might represent an ancestral feature which may contribute to the adaptation of genes relocated to the repressive environment without any deleterious impact on fitness.

To address these questions, we analyzed a representative set of more than 30 genes that reside in the pericentric heterochromatin of both arms of chromosome 2 in *D. melanogaster,* while in *D. virilis* the orthologs of these genes are located in different euchromatic regions of the same chromosomal elements (Table S2)^21^. Genes demonstrating the opposite scenario of relocation in these two species were also included in the analysis. Among them we considered as heterochromatic the genes *Stim* and *Rrp47* that are located on the same scaffold_13050 as *Myb* and *Ranbp16* of *D. virilis,* as well as the genes *RpL15*, *Calr*, *Atg2* and *CG40228* that are embedded in scaffold_12736 located near the chromocenter of *D. virilis* (Table S2)^18^. In contrast to *D. virilis*, most of these genes, with the exception of *RpL15* and *CG40228*, are located in euchromatic regions of different chromosomal elements in *D. melanogaster* (Table S2). It is of note that localization of all of the selected genes was confirmed by in situ hybridization technique in this and other studies^18,21^. Also, due to the low annotation of 5′UTRs in *D. virilis* we have extended the analyzed region to 10 Kb upstream the annotated gene loci in order to identify the nearest binding of BEAF-32.

**Figure 4:**
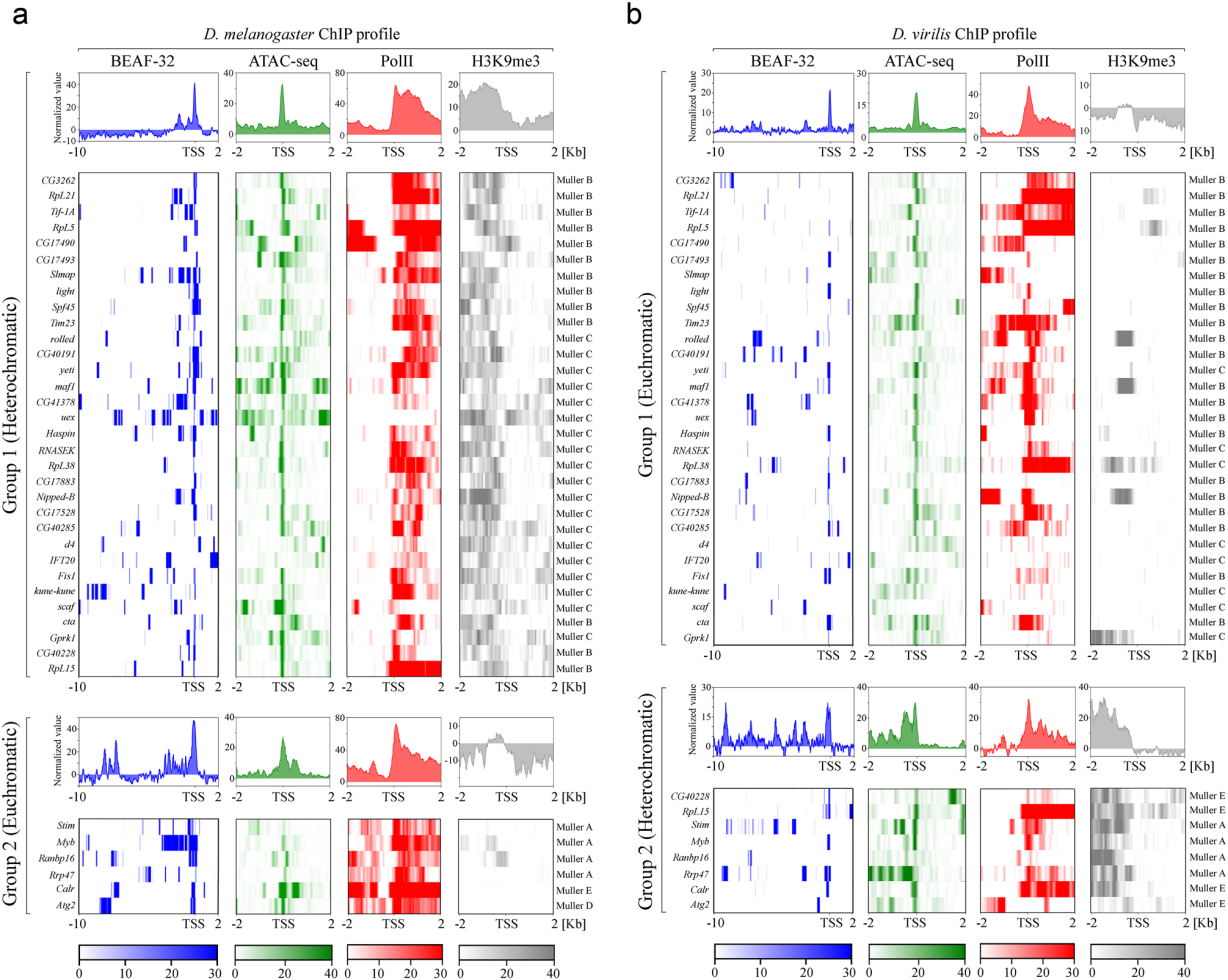
Enrichment of BEAF-32 near the TSS of genes repositioned between euchromatin and heterochromatin during *Drosophila* evolution. (**a**) and (**b**) show the enrichment profiles of ChIP-seq reads of genic loci of *D. melanogaster* and *D. virilis*, respectively. Note that the analyzed area for BEAF-32 enrichment includes 10 Kb upstream and 2 Kb downstream from TSS, while for RNA Pol II, H3K9me3 and ATAC-seq reads area includes 2 Kb both upstream and downstream from the TSS. Color codes below the heatmaps indicate fold enrichment (treat vs input).

Using these sets of genes and specified parameters, we performed enrichment analysis of BEAF-32, Pol II, H3K9me3 and ATAC-seq reads upstream and downstream of the TSS in all of these genes (Fig. 4). Given the opposite chromatin state of the studied genes, we subdivided gene sets into two groups, group 1 includes genes that reside in heterochromatic regions in *D. melanogaster* but located in euchromatin in *D. virilis* (Fig. 4)*.* Group 2 consists of genes located within euchromatin in *D. melanogaster* and heterochromatin in *D. virilis*. As indicated in Fig. 4, the insulator protein BEAF-32 is highly enriched in the vicinity of TSS of virtually all considered heterochromatic genes in both *D. melanogaster* (group 1) and *D. virilis* (group 2) (Fig. 4). However, binding of BEAF-32 in the promoters of heterochromatic genes does not necessarily coincide with the TSS of their euchromatic orthologs (group 1 of *D. virilis* and group 2 of *D. melanogaster*) (Fig. 4, Table S2). Overall, we observed 27/38 shared genes comprising both groups (euchromatic and heterochromatic) of *D. virilis* and *D. melanogaster* which exhibit similar BEAF-32 binding in their promoters (Fig. 4, Table S2). The binding area of BEAF-32 is in strong association with the enrichment profile of Pol II and ATAC-seq reads in the proximity of TSS for most of the studied genes (Fig. 4). Importantly, the mean enrichment value of BEAF-32 in the vicinity of the promoter does not differ significantly between heterochromatic genes (median: 8) and euchromatic ones (median: 9.25) with P = 0.4 (Mann–Whitney U test) indicating that the enrichment of BEAF-32 does not depend on the local chromatin environment.

Motif CGATA is a hallmark of BEAF-32 genomic binding sites^41,42^. However, recently it was shown that even though BEAF-32 can bind DNA directly, a large subset of BEAF-32 peaks that does not share BEAF-32 consensus motif and apparently mediates functional long-range contacts among distinct insulator sites through indirect binding with a co-factor CP190^43^. Given this, we have analyzed group 1 and group 2 genes and observed that 27/32 of heterochromatic genes and 4/6 of euchromatic genes in *D. melanogaster* comprising group 1 and 2, respectively, share motif CGATA in their promoters (within 200 nt upstream from TSS) demonstrating the direct binding of BEAF-32, while others do not and thus exhibit the indirect binding of BEAF-32 (Table S2). In *D. virilis* we have defined 15/30 of euchromatic genes (group 1) and 8/8 of heterochromatic genes (group 2) exhibiting a direct binding of BEAF-32. In agreement with the previous findings^43^, direct peaks exhibit greater enrichment than indirect ones (median of direct peaks: 11, median of indirect peaks: 5, P < 0.01 by Mann–Whitney U test).

Taken together, these results indicate that BEAF-32 binds predominantly directly the promoters of genes that were juxtaposed with heterochromatin during the evolution of the genus Drosophila. However, while the promoters of heterochromatic genes of *D. melanogaster* are occupied by BEAF-32, their euchromatic orthologs in *D. virilis* have partially lost their BEAF-32 binding sites in the vicinity of promoter regions.

### Promoters of most heterochromatic genes are occupied by insulator proteins BEAF-32, GAF and dCTCF in *D. melanogaster*

It is known that among the described insulator proteins, BEAF-32, GAF and dCTCF are widespread upstream of the TSS of actively transcribed genes in the genome of *D. melanogaster*^44–48^. Indeed, distribution of BEAF-32, GAF and dCTCF has a similar pattern in terms of genomic features (Figs. S4–S6). Most of the binding sites of these insulator proteins are located predominantly within 1 Kb upstream the TSS (BEAF-32 ~ 64% of all sites, GAF ~ 66% and dCTCF ~ 60%). Notably, these proteins also bind intronic (BEAF-32 ~ 7% of all sites, GAF ~ 7% and dCTCF ~ 8%) and intergenic (BEAF-32 ~ 10% of all sites, GAF ~ 6% and dCTCF ~ 17%) regions in *D. melanogaster* genome (Figs. S4–S6).

**Figure 5:**
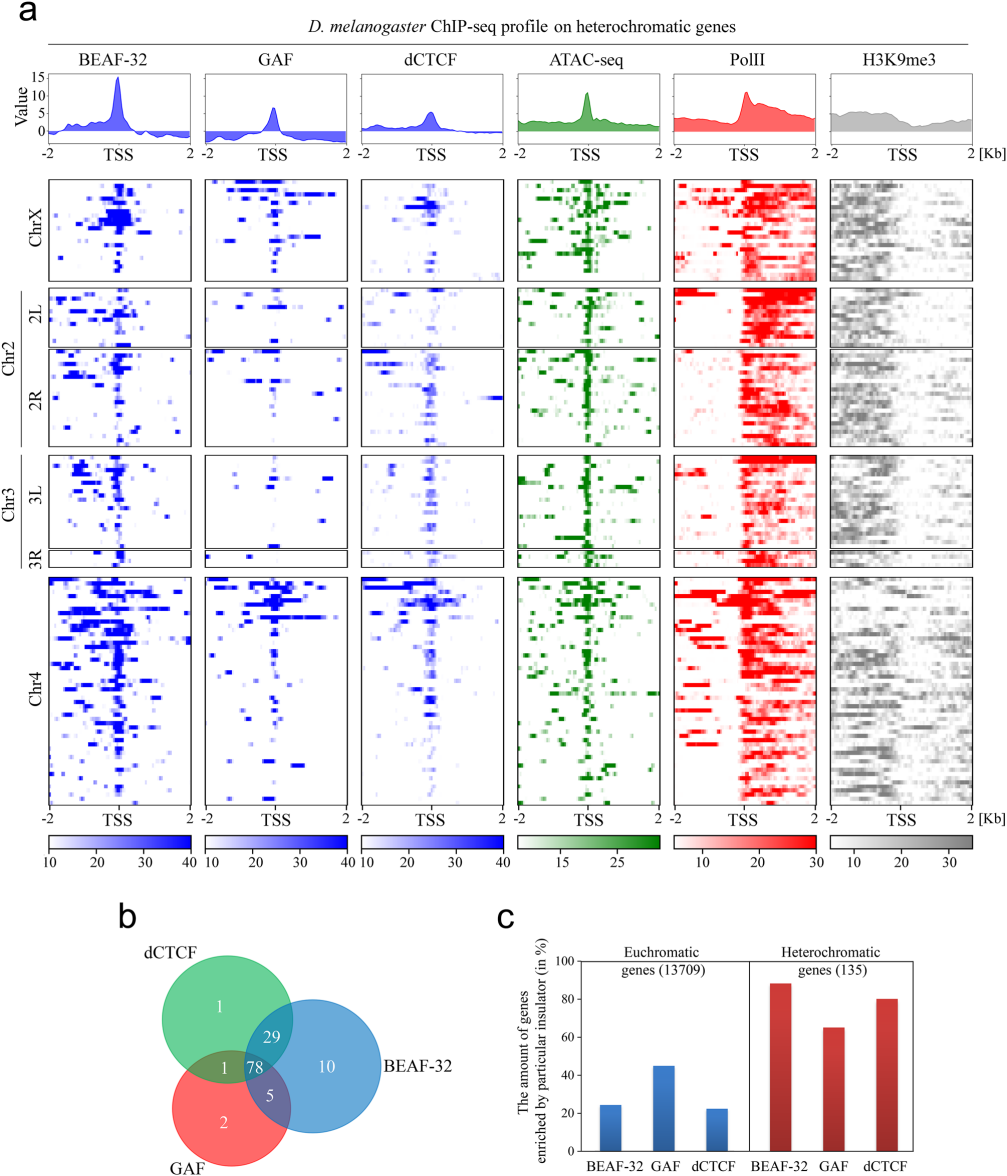
The enrichment profile of insulator proteins around the promoter regions of heterochromatic genes in *D. melanogaster*. (**a**) The enrichment profiles of ChIP-seq reads for BEAF-32, GAF, dCTCF, RNA Pol II, H3K9me3 and ATAC-seq reads on heterochromatic genes of *D. melanogaster*. The analyzed area includes 2 Kb upstream and 2 Kb downstream from TSS. Color codes below the heatmaps indicate fold enrichment (treat vs input). (**b**) Venn diagram indicates the number of gene loci enriched with all three, two and only one studied insulator protein BEAF-32, GAF and dCTCF. (**c**) Diagram shows the percentage of genes whose promoters are enriched with BEAF-32, GAF or dCTCF. Euchromatic and heterochromatic genes are indicated separately according to the number of genes comprising these regions.

In order to elucidate whether the promoters of all heterochromatic genes are occupied by insulator proteins, we analyzed the enrichment profile of BEAF-32, GAF and dCTCF within 2 Kb upstream and downstream of TSS of all heterochromatic genes of *D. melanogaster* (Fig. 5a). For this purpose, we sorted pericentric protein-coding genes located downstream of the euchromatin-heterochromatin border defined by a gradual increase of H3K9me3 and HP1a enrichment in *D. melanogaster*. We have also included genes from the dot chromosome 4 considering it as entirely heterochromatic^5^. Next, we selected only those genes that show significant enrichment of RNA Pol II and expression level not less than 10 RPM (reads per million), according to ChIP-seq and RNA-seq data, respectively. A final list of heterochromatic protein-coding genes includes 135 genes located at different chromosomes of *D. melanogaster* (Table S3).

The performed analysis of the enrichment of insulator proteins shows that although BEAF-32 occupies the majority of gene promoters it is not present ubiquitously (Fig. 5a). However, the lack of BEAF-32 near the promoters of heterochromatic genes is usually compensated by the presence other insulator proteins GAF and dCTCF, keeping the promoters of > 93% (126/135) heterochromatic genes enriched with insulator proteins (Table S3). Importantly, the binding area of insulator proteins is strongly correlated with the area of decreased levels of methylated H3K9 and nucleosome-free regions defined by ATAC-seq, which can be seen at a distance of 2 Kb and 1 Kb around TSS (Fig. S7). Notably, all three studied insulator proteins are present together in more than a half of the promoters of heterochromatic genes (78/135 genes, range of the promoter was defined as 200 nt upstream the TSS) (Fig. 5b). A lot of genes are occupied by a pair of the insulator proteins, e. g. BEAF-32 and dCTCF (29 genes) or BEAF-32 and GAF (5 genes) (Fig. 5b). Among the number of BEAF-32 binding sites, ~ 84% (104/121 genes) share CGATA motif indicating the predominantly direct binding of this insulator proteins to the promoters of heterochromatic genes. Consistent with the previous observation, the enrichment of BEAF-32 in direct peaks are ~ 2.5 fold greater than in indirect ones (median of direct peaks: 23, median of indirect peaks: 11, P < 0.01, Mann–Whitney U test). Notably, there is a small subset (9 of 135) of heterochromatic genes that lack the studied insulator proteins in their promoters. Part of them (*CG17450*, *CG33502*, *CG32857, CG32820*, *CG32819,* and *CG32500*) are located within a gene cluster which includes eight closely spaced genes and covers 30 Kb of the genome (Fig. S8). Such gene cluster is not typical for the pericentric heterochromatin where genes are widely spaced by repetitive sequences. Interestingly, the promoters of the flanking genes (*GCS2α* and *DIP1*) of this cluster are occupied by the studied insulator proteins (Fig. S8). It can be speculated that such a placement could allow the formation of a loop between the flanking genes to form an actively transcribed region, which eliminates the need to keep insulators in the promoters of each gene included in this structure.

It is evident that insulator proteins occupy a higher number of genes in heterochromatin than euchromatin (Fig. 5c). Specifically, BEAF-32 is the most prominent insulator that is enriched in the promoters of heterochromatic genes (> 90% (122/135) of all heterochromatic genes), while GAF is prevalent in euchromatic genes (49% (6,717/13,709) of all euchromatic genes) (Fig. 5c).

Taken together, these data show that insulator proteins BEAF-32, GAF and dCTCF, solely or in combination with each other, are present in the promoters of virtually all heterochromatic genes of *D. melanogaster* studied so far.

### BEAF-32 and Dref binding overlaps in the promoters of a subset of heterochromatic genes implicated in gene transcription in *D. melanogaster*

**Figure 6:**
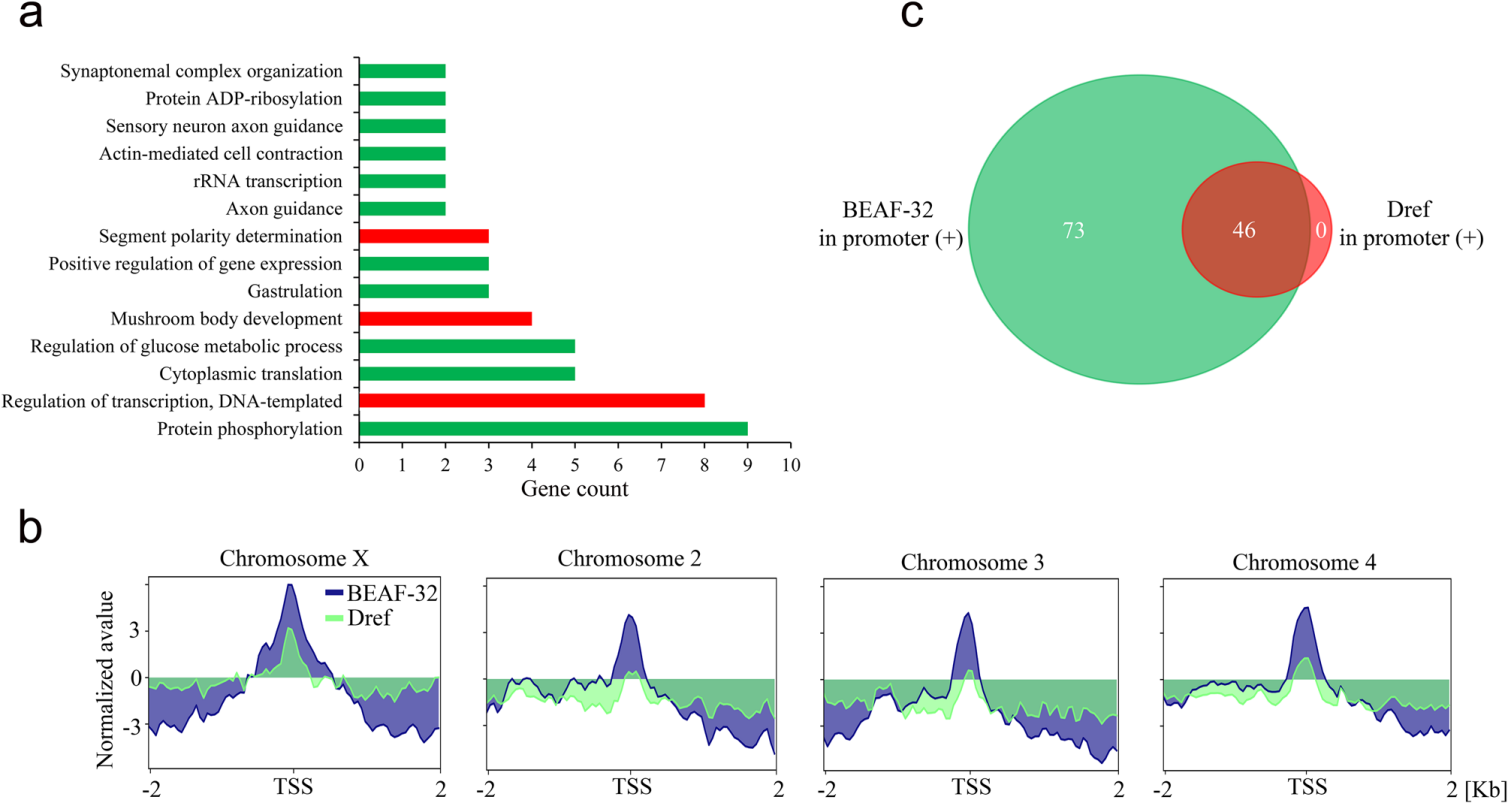
Enrichment of BEAF-32 and Dref in the promoters of heterochromatic genes in *D. melanogaster*. (**a**) Gene Ontology (GO) analysis by biological processes of all heterochromatic genes (P < 0.05 of presented GO terms). Green color bars denote the processes in which genes occupied by BEAF-32 are involved, red bars: genes enriched with BEAF-32 and Dref. (**b**) Enrichment plots of BEAF-32 and Dref in heterochromatic genes of *D. melanogaster*. Plots for heterochromatic genes are given separately for each chromosome. (**c**) Venn diagram indicates the number of heterochromatic genes whose promoter regions are occupied solely by BEAF-32 or Dref as well as overlapped genes.

Dref is described as a master regulator of cell proliferaton in Drosophila^49^. The DNA recognition motif for BEAF-32 (CGATA) is contained within Dref binding sequence (TATCGATA), and the binding of Dref to DNA has been shown to antagonize the binding of BEAF-32 in vitro^50^. In order to demonstrate to what extent BEAF-32 binding overlaps with Dref in the promoters of heterochromatic genes, we have analyzed the enrichment of these proteins in the promoters of previously defined heterochromatic genes of *D. melanogaster* using ChIP-seq data from Kc167 cell line (see “Methods”). Notably, genes embedded into heterochromatin in *D. melanogaster* are implicated in a variety of biological processes that mediate many aspects of cellular function, including protein phosphorylation, transcription, translation, development and recombination processes (Fig. 6a).

Enrichment analysis shows that BEAF-32 binds DNA within the promoters of 88% (119/135) of heterochromatic genes in Kc167 cell line (Table S4). Among them, 102 genes exhibit CGATA motif within their promoter regions indicating the predominantly direct binding of BEAF-32 to the heterochromatic gene loci as was indicated previously (Table S4, Fig. 5). In contrast to BEAF-32, the enrichment of Dref is significantly lower in the promoters of heterochromatic genes of *D. melanogaster* (Fig. 6b). Thus, binding of Dref in the promoters of heterochromatic genes overlaps with BEAF-32 for 46 genes among 119 genes occupied by BEAF-32 (Fig. 6c, Table S4). Most of these genes (e.g. *pan*, *hcf*, *Sox102F*) are implicated in regulation of transcription and developmental processes (red bar in Fig. 6a). Moreover, in contrast to BEAF-32, the binding of Dref does not occur in the absence of BEAF-32 (Fig. 6c). Importantly, a half (23/46) of genes those promoters are occupied by Dref do not share the canonical Dref motif TATCGATA (Table S4) indicating that Dref binding may be weaker in the promoters of heterochromatic genes. Indeed, the overall enrichment of BEAF-32 is 3–fourfold higher than Dref (median of BEAF-32 peaks: 21, median of Dref peaks: 5, P < 0.01 by Mann–Whitney U test). Notably, there are 16 genes involved in developmental processes occupied neither by BEAF-32 nor by Dref in Kc167 cell line (Table S4).

These data indicate that Dref is present in the promoters of heterochromatic genes together with BEAF-32, suggesting that BEAF-32 may be required for Dref binding to the promoters of these genes.

### Disruption of BEAF-32 has a complex effect on genome expression affecting even those genes that lack this insulator protein in their promoter regions

As seen from the results above, BEAF-32 is the most prevalent insulator protein in promoters of genes in the pericentric heterochromatin in *D. melanogaster*. Drosophila *BEAF-32* gene encodes two isoforms, BEAF-32A and BEAF-32B. Both proteins are essential, but BEAF-32B alone is sufficient for the viability of flies^51^. Homozygous mutation of BEAF32 is characterized by disorders in oogenesis, resulting in drastically reduced fertility of females^51^. To find out to what degree BEAF-32 contributes to expression of genes, we analyzed RNA-seq data of stably transfected *Drosophila* Schneider S2 cells expressing mutant BEAF-32 in the absence of endogenous protein^43^.

**Figure 7:**
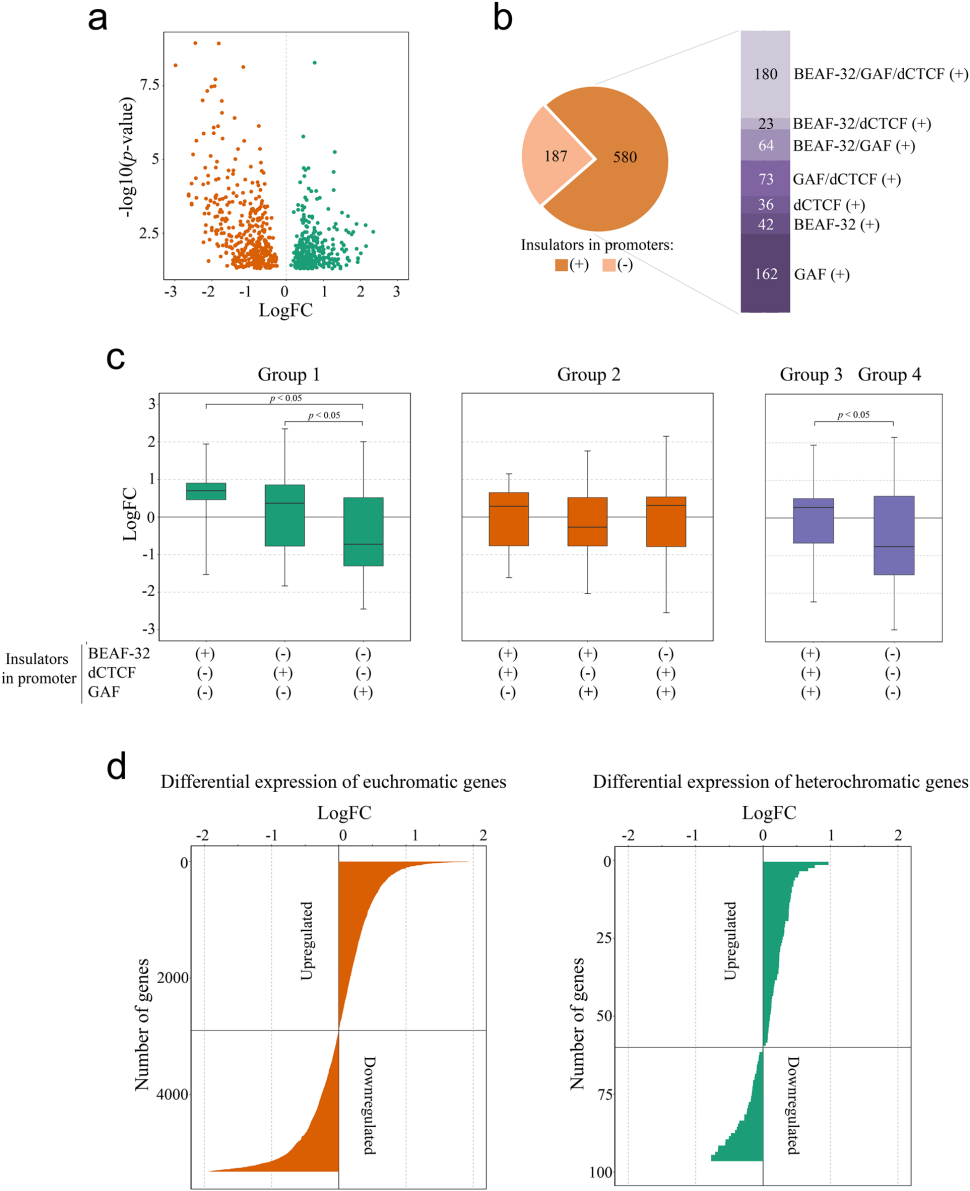
Mutant BEAF-32 strongly affects gene expression both in euchromatin and heterochromatin in *D. melanogaster*. (**a**) Volcano plot demonstrating genes with expression changes in *Drosophila* S2 cells expressing mutant BEAF-32 in comparison with control cells (767 genes with P ≤ 0.05). Genes with expression level < 1 Log10 CPM (counts per million) were discarded. (**b**) Pie charts show the number of differentially expressed genes whose promoters are enriched with one, two or simultaneously occupied by all three insulator proteins BEAF-32, dCTCF and GAF (580 differentially expressed genes, P ≤ 0.05). Genes whose promoters are free of these insulator proteins are also shown (187 differentially expressed genes, P ≤ 0.05). (**c**) Box plots demonstrate trends of gene expression changes upon the impairment of BEAF-32 function. All differentially expressed genes (P ≤ 0.05) are divided into 4 groups according to the presence of insulator protein in their promoter regions—group 1 (one of three proteins), group 2 (combination of two of three proteins), group 3 (combination of all three insulator proteins) and group 4 (genes that are not occupied by these proteins). *Indicates P ≤ 0.05 (Fisher exact test). d) Bar plots demonstrate overall expression changes in S2 cells expressing mutant BEAF-32 separately for euchromatic genes (left) and heterochromatic genes (right).

The analyzed data indicate that impairment of BEAF-32 strongly affects gene expression (767 differentially expressed genes (DEG), P ≤ 0.05; Fig. 7a). Notably, the disruption of BEAF-32 function has a complex effect on transcription, which includes not only downregulation but also upregulation of gene expression levels (Fig. 7a). Given that insulator proteins BEAF-32, dCTCF and GAF may overlap in promoters of genes, we sorted DEG according to the association of these insulators with DEG. We found that among 580 DEG the largest groups comprise all three insulator proteins (180 DEG), and 162 DEG contain only GAF (Fig, 7b), whereas BEAF-32-associted DEG include only 42 genes. Furthermore, 64 and 23 DEG exhibit overlapping with GAF and dCTCF, respectively (Fig. 7b). Interestingly, among differentially expressed genes 187 genes (P ≤ 0.05) are not occupied by any of the three proteins (BEAF-32, dCTCF and GAF) (Fig. 7b).

Next, we estimated trends of gene expression changes analyzing separately DEG showing association with BEAF-32, dCTCF, and GAF or a combination of these proteins. Unexpectedly, BEAF-32 associated DEG tend to be upregulated upon disruption of BEAF-32 function (left box in group 1, Fig. 7c). On the other hand, downregulation is observed for GAF-associated genes (right box in group 1, Fig. 7c) and for the genes that are not occupied by any of the studied insulator proteins (group 4, Fig. 7c). Other groups of genes, in particular the ones associated with dCTCF only (middle box in group 1), a pair of insulator proteins in various combinations (group 2) and DEG associated with all three insulator proteins (group 3) exhibit a complex pattern of gene expression, with upregulated and downregulated changes (Fig. 7c). Moreover, the upregulated expression profile of BEAF-32 and dCTCF-associated genes are significantly different from GAF-associated DEG (*p* ≤ 0.05). The same applies to DEG that are associated with all three proteins in comparison with genes that are not occupied with any of them (group 3 vs group 4, P ≤ 0.05).

Overall, effect of mutation disrupting BEAF-32 is more pronounced for euchromatic genes than heterochromatic ones (Fig. 7d). Among euchromatic genes more than 25% (P ≤ 0.05) were affected, while only 15% of heterochromatic genes show expression changes higher than 1.5 fold (P ≤ 0.05, Fig. 7d), suggesting that expression of heterochromatic genes is more robust to the disruption of the function of only one of the insulator proteins.

Therefore, these data show that the impairment of BEAF-32 strongly affects expression of both euchromatic and heterochromatic genes, including those that lack this insulator protein in their promoter regions.

## Discussion

During speciation, the genomes of *Drosophila* species underwent multiple chromosome rearrangements that disrupt gene order, modifying or preserving gene function^17,52^. In this study, we show that in the course of evolution of *Drosophila* species the essential *Myb* and *Ranbp16* genes have been relocated into different chromatin types, i.e. euchromatin or heterochromatin. Despite the contrasting chromatin structure and local repressive environment of heterochromatic regions enriched with repetitive DNA, both genes were shown to be under purifying selection due to their highly conserved and vital function.

According to studies of PEV, genes that are juxtaposed with heterochromatin by chromosomal rearrangements or transposition events exhibit a variegating phenotype resulting in silencing of genes due to heterochromatin environment^8^. Given that, how might the evolutionary relocation of essential genes into the pericentric heterochromatin be explained? What determines the insusceptibility of regulatory regions of heterochromatic genes to the repressive surroundings? Considering the peculiarities of heterochromatic genes, such as accumulation of TEs within their introns, one may suggest that heterochromatic genes have evolved to adapt to the heterochromatic environment and became dependent on heterochromatin specific proteins^4,53–56^. Previously, it was shown that functioning of heterochromatic genes depends on repetitive environment and heterochromatin factors, such as HP1a, to facilitate their expression and probably long-distance interactions between enhancers and promoters^11,57–59^. Moreover, evolutionary relocation preferentially occurred only with genes exhibiting close association with HP1a, suggesting that HP1a binding to these genes existed in the progenitor^21^. An analysis conducted by Yasuhara et al.^20^ demonstrated that promoters of heterochromatic genes have not undergone major alterations after relocation into the repetitive environment of heterochromatin, which excludes the existence of heterochromatin-specific promoters. Together, these observations allow one to suggest that certain gene loci were probably pre-adapted or had acquired adaptive properties in the ancestral species for relocation between euchromatin to heterochromatin in the course of evolution. If this is the case, gene loci relocated to heterochromatin probably should retain the transcriptionally active euchromatin-like structure of chromatin capable of efficient transcription in the heterochromatin. Indeed, the proximal regulatory regions of heterochromatic genes are not occupied by the heterochromatic mark H3K9me3, forming a nucleosome-free binding platform for transcriptional factors and RNA polymerase II. Recently, a remarkable peculiarity of HP1a binding at several gene loci has been described, whereby HP1a can be recruited to gene promoters independently of H3K9 methylation^60^. Along these lines, the observed binding of BEAF-32 in the vicinity of gene promoters which underwent relocation between euchromatin and heterochromatin in evolutionarily distant species of *Drosophila* is very intriguing. In cooperation with HP1a, the presence of BEAF-32 and probably other insulator proteins such as dCTCF and GAF at gene promoters probably contributed to the formation of “proto-heterochromatic” gene loci in the ancestral species of *Drosophila* and thus facilitated their normal functioning in the heterochromatic environment. Hence, one may hypothesize that insulator proteins may block the spreading of heterochromatin to the promoter regions of heterochromatic genes, while HP1a maintains a proper chromatin structure at and around such gene loci that might be controlled epigenetically.

Originally, insulator proteins were defined as regulators of interaction between enhancers and promoters able to block silencing effect of PEV^22–25^. A growing body of evidence suggests that insulator proteins exercise diverse roles, including barrier function, and mediate short and long-distance chromosomal contacts at the genome-wide scale^45,61–64^. Our enrichment analysis of ChIP-seq data indicated that the insulator protein BEAF-32 is enriched upstream of the TSS of heterochromatic genes in *D. melanogaster* and *D. virilis,* probably demarcating the euchromatin-heterochromatin border between the promoter and the surrounding heterochromatin. Furthermore, using the same set of genes that reside in different types of chromatin in *D. melanogaster* and *D. virilis,* we show that BEAF-32 binding is predominantly preserved in the promoter regions of heterochromatic genes during evolution of different *Drosophila* species, suggesting that BEAF-32 binding is an ancestral property of these genes, rather than adaptation to the heterochromatic environment. However, the binding of BEAF-32 in the vicinity of TSS is not always preserved in euchromatic genes in comparison to their heterochromatic orthologs in distant species (Fig. 4). One may suggest that due to the high density of genes in euchromatic regions of the genome and lower occupancy of repressive histone mark H3K9me3, not every gene requires barrier insulation of BEAF-32 for functioning. In contrast to euchromatic regions, genes in the pericentric heterochromatin are largely dispersed, separated by numerous TEs and other repeats in intergenic regions enriched with H3K9me3 and HP1a. In such an environment barrier function of insulator proteins might have become one of the major determinants that contribute to the proper function of heterochromatic genes. Importantly, the binding sites of BEAF-32 near the promoters could be indirect even in the heterochromatic regions and result in weaker binding of insulator protein. As was shown previously, insulator proteins BEAF-32 and dCTCF may facilitate long-range contacts of the chromatin through CP190^43^. However, how this machinery works remains an unresolved question. Obviously the interactions of cis-regulatory elements and trans-acting factors involved are more complex and include a variety of chromatin-remodeling factors and insulators acting to facilitate continuous gene expression and chromosomal architecture of heterochromatic gene loci.

Along with BEAF-32, insulator proteins dCTCF and GAF are also enriched at the TSS of heterochromatic genes. Moreover, a combination of BEAF-32, GAF and dCTCF covers virtually all promoters of protein-coding genes located in the pericentric chromosome regions and heterochromatic dot chromosome 4 in *D. melanogaster,* suggesting that proper functioning of heterochromatic gene loci requires insulators (Fig. 5). However, there is a subset of genes whose promoters lack the binding site of these three insulator proteins suggesting that their functioning is mediated by other insulator proteins such as Pita that also belongs to a class of insulator proteins that preferentially bind to promoters near the TSS^65^. Alternatively, the architecture of certain gene loci especially of gene clusters probably allows functioning without these regulatory elements resulting in their eventual loss in the promoter regions during evolution.

The insulator proteins GAF and especially dCTCF have plenty of overlapping binding sites with BEAF-32 and Dref in the *Drosophila* genome^66^. Moreover, Dref co-localizes at the same genomic sites as BEAF-32 and other insulator proteins and is enriched at the boundaries of topologically associated domains (TAD)^66^. To this end, we observed that the promoters of heterochromatic genes do not appear to have Dref without binding BEAF-32 (Fig. 6). Notably, *cis*-acting elements that exercise the transcriptional control of genes by Dref, as well as protein sequence of Dref, are conserved between such evolutionarily distant species as *D. melanogaster* and *D. virilis *^46,67^. Together, these data probably suggest that Dref function in heterochromatin is mediated by and might depend on insulator proteins on an evolutionary timescale.

It is of note that a direct impact of insulator presence on gene expression has been established for the *D. melanogaster* GAGA factor (GAF) that resides in the *hsp70* promoter. GAF mediates the recruitment of chromatin remodeling factors, including SWI/SNF, the CHD, and the ISWI family complexes, that ensure formation of nucleosome-free region in the *hsp70* promoter^68–70^. Knockout mutation showed that BEAF-32 is important for both oogenesis and development^51^. Furthermore, it was shown that PEV of the *w*^*m4h*^ allele and different *y* transgenes was enhanced by the BEAF-32 KO, suggesting that BEAF-32 function affects chromatin structure or dynamics^51^. Other studies of BEAF-32 demonstrated that most BEAF-associated genes are transcriptionally active or even highly expressed and are associated with negative elongation factor Nelf that stimulates transcription levels by inhibiting promoter-proximal nucleosome assembly^61,71^. This provides evidence that BEAF-32 facilitates high levels of gene expression. Indeed, the mutation of BEAF-32 which abrogates BEAF-32 function results in misregulation of hundreds of genes^43^. Surprisingly, most of the affected genes show an association predominantly with GAF (162 genes) but not with BEAF-32 (42 genes) (Fig. 7). Moreover, the downregulation of gene expression was observed mostly for genes that lack direct association with BEAF-32 protein. According to this complex pattern of gene expression, one may suggest that deficiency of BEAF-32 disrupts chromosomal contacts, resulting in misregulation of genome-wide expression.

While it is clear that further studies are needed to elucidate all the factors required for normal gene functioning in the heterochromatic surroundings, our results suggest a possible evolutionary path that can be utilized by heterochromatic genes to maintain their expression in the repressive environment.

## Conclusions

Heterochromatin in *Drosophila* is generally associated with transcriptional silencing. Nevertheless, dozens of essential genes have been identified in the pericentric heterochromatin of *D. melanogaster* and other species. In this study, we investigated the molecular evolution of the essential genes that were relocated between euchromatin and pericentric heterochromatin in the phylogeny of *Drosophila*. By surveying factors necessary for normal functioning of genes relocated into heterochromatin in distant *Drosophila* species, e.g. *D. melanogaster* and *D. virilis*, we conclude that certain insulator proteins (i.e. BEAF-32) may contribute to the successful adaptation of genes to the pericentric heterochromatin by facilitating normal gene expression in the repressive surrounding.

## Methods

### Drosophila genomes and sequence analyses

Drosophila genomes and gene sequences for comparative analysis were extracted from FlyBase and NCBI databases. Sequences of genomic regions containing *Myb* gene of virilis group species (*D. lacicola, D. littoralis, D. borealis, D. flavomontana, D. lummei, D. ezoana)* were fetched from unpublished data of Dr. Venera Tyukmaeva and Prof. Michael Ritchie from the University of St. Andrews, UK (personal communication). Orthologous genes were retrieved from OrthoDB v9.1^72^. In the case of absence of gene annotation (e.g. for *D. guanche*, *D. subobscura*, most of the virilis group species), orthologs were retrieved with TblastN^73^ using protein sequence of the most closely related species (i.e. *D. obscura* for *D. subobscura* and *D. guanche*, *D. virilis* for *D. lacicola* and other virilis species) as queries. All the query subjects mapped on the same DNA strand adjacent to each other with E-value > e-80 were considered as valid. To perform reciprocal BLAST, obtained hits were aligned back to the original genome. Aligned hits were considered as the best reciprocal hits and used for reconstruction of coding sequences. Sequences between mapped subjects were considered as introns. Putative transcriptional start sites (TSS) of poorly annotated genes were identified with blastN^74^ using 1st exon sequence of related species. Blast results with E-value > e-60 and adjacent to annotated coding sequence at a distance not exceeding 600 nt were considered as true and the 1st mapped nucleotide as TSS. All essential information, including genes IDs, genomes IDs, and genomic coordinates of *Myb* and *Ranbp16* in all studied species, is listed in Table S1. Orthologous sequences of *Myb* and *Ranbp16* genes of *Anopheles gambiae* were extracted from VectorBase (https://www.vectorbase.org/) by the numbers AGAP008160 – *Myb* and AGAP004535 – *Ranbp16*. Protein sequences of *Myb* and *Ranbp16* (also known as *Xpo7*) of mouse and human were extracted from UniProt (https://www.uniprot.org/). Protein motifs were scanned using the PROSITE database and methodology^75,76^*.*

Estimation of repeat content in intergenic regions and within studied genes was performed using RepeatMasker (https://www.repeatmasker.org) and computationally predicted libraries of TEs generated with ReAS^77^ that are available in FlyBase (ftp://ftp.flybase.net/genomes/aaa/transposable_elements/ReAS/v2/consensus_fasta/). For repeat masking of *D. miranda* genome, we used consensus sequences of TEs of *D. pseudoobscura* and *D. persimilis*, and for *D. hydei* we applied the library of *D. mojavensis*. TEs were classified using RepeatClassifier implemented in RepeatModeler software^78^.

Multiple sequence alignment was performed with ClustalW^79^ and Clustal Omega^80^ programs (https://www.ebi.ac.uk/Tools/msa/) for nucleotide and amino acid alignments, respectively. Multiple protein alignments were visualized with Jalview^81^.

Circular plot was made using Circos visualization tool^82^. Flanking regions of *Myb* and *Ranbp16* (20 Kb upstream and downstream from the gene location) were used instead of intergenic regions, due to the long distance between these genes in *D. miranda* (> 17 Mbp) and low scaffold contiguity around these genes for *D. persimilis* and *D. hydei*.

### ChIP-seq, RNA-seq and ATAC-seq analyses

Raw data of genome binding/occupancy (ChIP-seq), transcriptome (RNA-seq) and nucleosome (ATAC-seq) profiling were obtained from GEO database and used in the analyses. They include: GSE59965—contains data for *D. virilis* including RNA-seq, ChIP-seq of H3K9me3 and RNA polymerase II performed using commercially available anti-H3K9me3 (ab8898, Abcam) and anti-RNA Pol II (ab5408, Abcam) antibodies; GSE35648—contains data for both *D. melanogaster* and *D. virilis* including ChIP of BEAF-32 performed using antibodies generated against amino acids 1–83 of the major highly conserved isoform BEAF-32B in *D. melanogaster*^42^; GSE43829—contains RNA-seq as well as ChIP-seq of H3K9me3 and RNA polymerase II for *D. melanogaster* performed using aforementioned commercially available antibodies ab8898 and ab5408; GSE56347—includes ChIP-seq of HP1a for *D. melanogaster* performed with polyclonal anti-HP1 (PRB-291C, Covance innovative); GSE102439—includes ATAC-seq data for *D. melanogaster* and *D. virilis*; GSE62904 – ChIP-seq for Dref and BEAF-32 in Kc167 cells of *D. melanogaster*; finally, GSE85404 and GSE70632—contains ChIP-seq data of dCTCF and GAF for *D. melanogaster*. Comparative analysis of each deep sequencing data was conducted on the same type of tissue of *D. melanogaster* and *D. virilis*.

For analysis of sequence data, we used genome sequence and annotations released in FlyBase, *D. melanogaster* r.6.19 and *D. virilis* r1.06. Prior to mapping, all libraries were subjected to adapter clipping, filtering by length (> 20 nt) and quality (80% of nt must have at least 20 Phred quality) using TrimGalore (https://github.com/FelixKrueger/TrimGalore). Then, sequences were aligned to corresponding genomes using Bowtie^83^ with the following settings: “–best –strata -m 1”, retaining only uniquely mapped reads. Output sequence alignment map (SAM) files were converted to binary (BAM) format using SAMtools^84^. Aligned reads normalized to input samples in wig format were visualized using the Integrative Genome Viewer (IGV)^85^.

Peak calling was performed using MACS software^86^ with the recommended parameters for narrow (PolII, BEAF-32, dCTCF, GAF) and broad peak calling (H3K9me3, HP1a) as well as normalization on input chromatin controls. Enrichment analysis was performed using pipelines implemented in deepTools package^87^ with the parameters including ignoring of duplicates.

ChIP-indirect and direct peaks of BEAF32 were identified as described in Liang et al.^43^. Briefly, identified peaks from MACS that overlap with promoter regions (200 nt upstream TSS) were scanned for DNA-binding motif of BEAF-32 (extracted from JASPAR database^88^) using TFBSTools package^89^. If motif exists, binding is considered as direct, and in the absence of appropriate DNA motif in peak, binding is considered indirect.

For ATAC-seq analysis, reads that mapped on mitochondrial genomes were discarded, and peak calling was performed using Genrich (https://github.com/jsh58/Genrich) with the following settings: “-j -y -r -d 50”, including removal of PCR duplicates.

The analysis of enriched gene ontology (GO) terms was performed using DAVIDWebService package for R with a P = 0.05 (Fisher exact test)^90,91^.

### Gene expression analysis of mutant BEAF-32 by RNA-seq

Raw data for stably transfected *Drosophila* Schneider S2 cell line expressing synthetic WT/mutant BEAF-32 in the absence of endogenous BEAF-32 were fetched from NCBI GEO (GSE52887)^43^. Processing of data included the adapter, length and quality trimming by Trimmomatic, mapping of reads to the genome (release GRCm38) by STAR aligner, counting the overlap of reads with genes by featureCounts, implemented in PPLine script^92–95^. Differential gene expression analysis was performed with the edgeR package using a Fisher exact test between experimental groups^96^. The genes with expression level ≥ 1 Log10 CPM (counts per million) and P ≤ 0.05 were considered as differentially expressed. Three biological replicates were analyzed for each sample.

Differential expression analysis, data visualization, and GSEA (Gene Set Enrichment Analysis) were performed using R project for statistical computing^91^. Visualization of experimental data was made with ggplot2 and GOplot R packages^97^.

### Promoter analysis

Because of insertion of DAIBAM MITE at a distance of 92 bp upstream from TSS of *Myb* in *D. virilis*, the promoter regions of *Myb* in studied species were shortened to 100 bp. After sequence extraction, promoter regions of *Myb* and *Ranbp16* were searched for common motifs using MEME^98^ and identification of matches to known transcription factors was performed by Tomtom^99^ using OnTheFly^38^ and REDfly v5.6^39,40^ databases implemented in MEME Suite 5.0.5^37^.

### Sequence evolution and testing for selection

Analysis of nucleotide substitutions per site was conducted in MEGA X^100^ using the Tamura-Nei model^101^. Rate variation among sites was modeled with a gamma distribution (shape parameter = 1). All positions containing gaps and missing data were eliminated (complete deletion option).

Ratio of nonsynonymous and synonymous substitutions (dN/dS) was estimated using PAL2NAL software^102^ by converting multiple sequence alignment of proteins and the corresponding nucleotide sequences into a codon alignment, and the calculation of synonymous (dS) and non-synonymous (dN) substitution rates using codeml program implemented in PAML package^103^.

### Cytology and DNA in situ hybridization

*D. virilis* larvae were grown at 18^0^C on standard medium supplemented with live yeast solution for 2 days before dissection. Salivary glands from 3^rd^ instar larvae were dissected in 45% acetic acid and squashed. DNA probes corresponding to *D. virilis Myb* (Dvir\GJ18431; FlyBase ID: FBgn0205590) were prepared by PCR using gene-specific primers (Forward_ GCAAGTGCGAGCACTGAAAA; Reverse_TGCATACTGAGGTGTGCCAG). Then, DNA probe was biotinylated by nick translation using Biotin-14-dATP (Thermo Fisher Scientific, USA) as described in^104^. Localization of the probe was made using the cytological map of *D. virilis* chromosomes^105^. Images were obtained by binocular microscope Nikon Alphaphot-2 YS2 (Japan).

### RNA isolation, RT-PCR and 5′-RACE analysis

Total RNA from 3rd instar larvae, adult females and gonads was isolated using Extract RNA reagent (Evrogen, Russia). Synthesis of the first strand of cDNA from total RNA and subsequent amplification of regions of interest were performed using MINT cDNA kit (Evrogen, Russia) following manufacturer’s instructions. For specific rapid amplification of cDNA 5′-end (5′-RACE) analysis, we applied two outward primers (primer1 5′-AGTAGTTGTGCGTAGCTGGA-3′; primer2 5′-GCTGCTTGCACAATGTTTCTA-3′) corresponding to the annotated 5′-fragment of *D. virilis Ranbp16* gene (Dvir\GJ18467; FlyBase ID: FBgn0205626). PCR reaction was conducted using Encyclo DNA polymerase (Evrogen, Russia). The resulting PCR fragments were cloned into pAL2-T vector (Evrogen, Russia) and sequenced using plasmid-specific primers. In all RT-PCR experiments, probes containing all components but lacking reverse transcriptase were used as negative controls. The obtained sequence of 5′UTR of *D. virilis Ranbp16* gene was deposited in GenBank under the number MN481598.

## Supplementary information

Supplementary file1 (TIF 1166 kb)

Supplementary file2 (TIF 1258 kb)

Supplementary file3 (TIF 3005 kb)

Supplementary file4 (TIF 903 kb)

Supplementary file5 (TIF 1024 kb)

Supplementary file6 (TIF 1008 kb)

Supplementary file7 (TIF 1338 kb)

Supplementary file8 (TIF 496 kb)

Supplementary file9 (DOCX 17 kb)

Supplementary file10 (XLSX 12 kb)

Supplementary file11 (XLSX 13 kb)

Supplementary file12 (XLSX 16 kb)

Supplementary file13 (XLSX 15 kb)

## Data Availability

The datasets supporting the conclusions of this article are available in the NCBI GEO repository: GSE59965; GSE35648; GSE43829; GSE56347; GSE102439; GSE85404; GSE70632; GSE36737; GSE62904; GSE52887. The obtained sequence of 5′UTR of *D. virilis Ranbp16* gene was deposited in GenBank under the number MN481598.
