# Supplementary figures and images for: Adaptation of gene loci to heterochromatin in the course of Drosophila evolution is associated with insulator proteins
Source: Sci Rep. 2020 Jul 17;10:11893. doi: 10.1038/s41598-020-68879-2 (PMC7368049; doi:10.1038/s41598-020-68879-2)

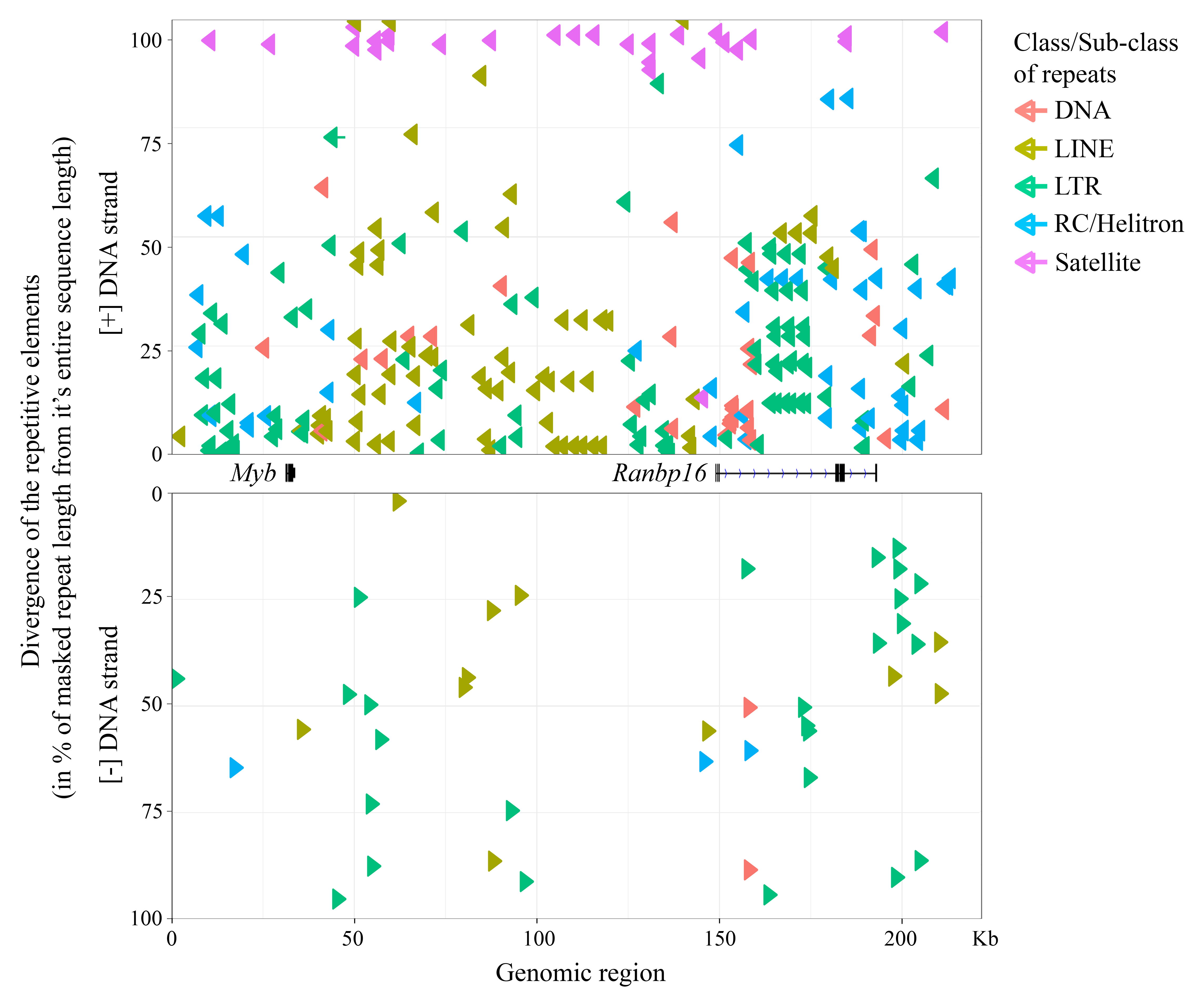

Supplement: Supplementary file 1 — Supplementary file1 (TIF 1166 kb) [file 41598_2020_68879_MOESM1_ESM.tif]

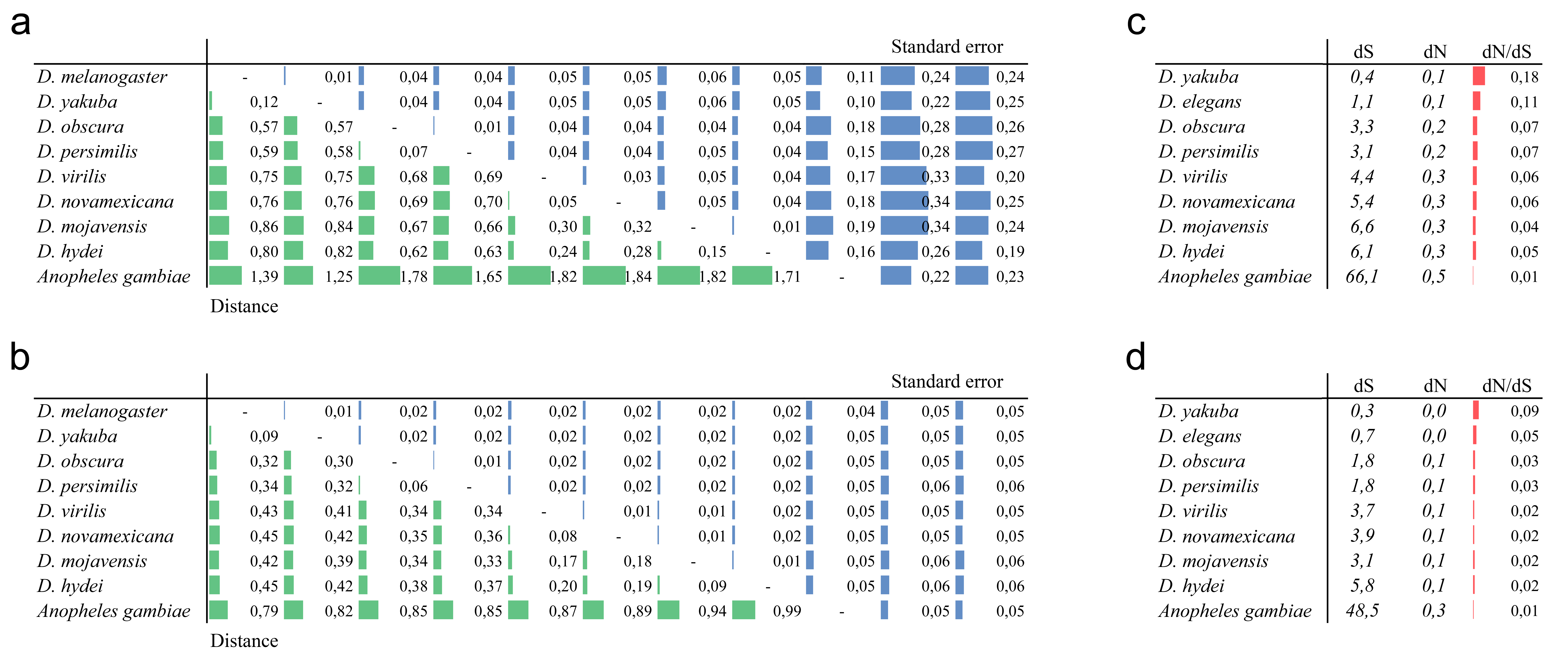

Supplement: Supplementary file 2 — Supplementary file2 (TIF 1258 kb) [file 41598_2020_68879_MOESM2_ESM.tif]

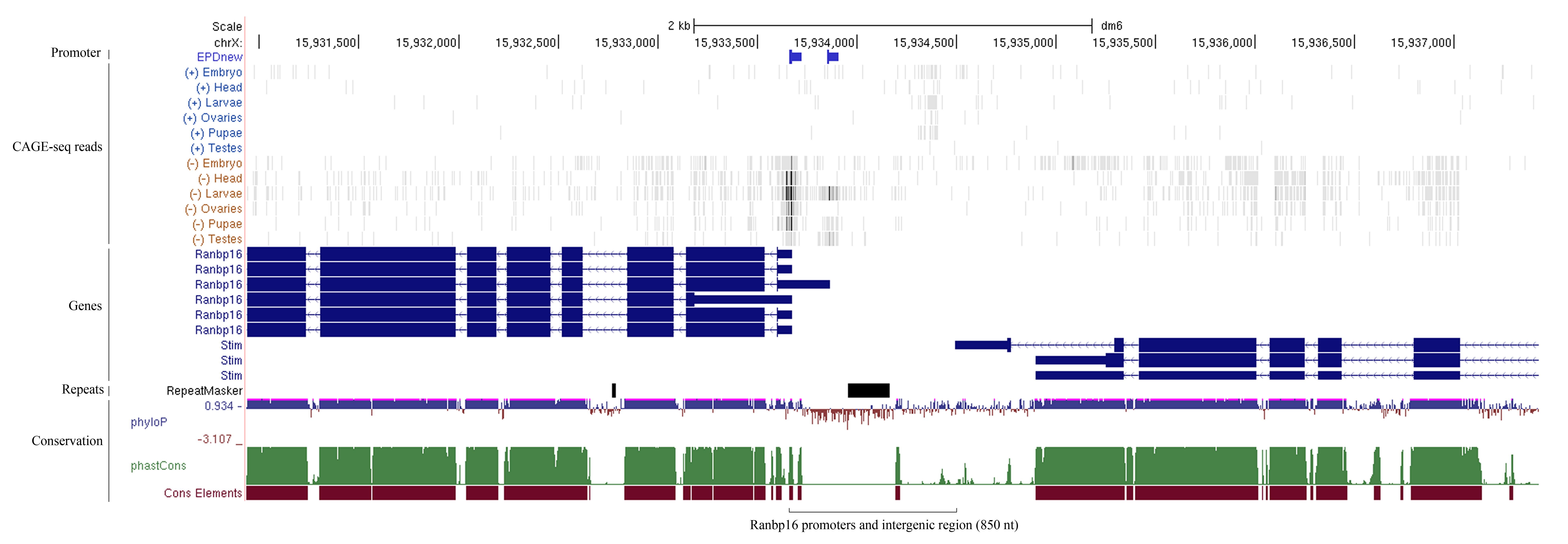

Supplement: Supplementary file 3 — Supplementary file3 (TIF 3005 kb) [file 41598_2020_68879_MOESM3_ESM.tif]

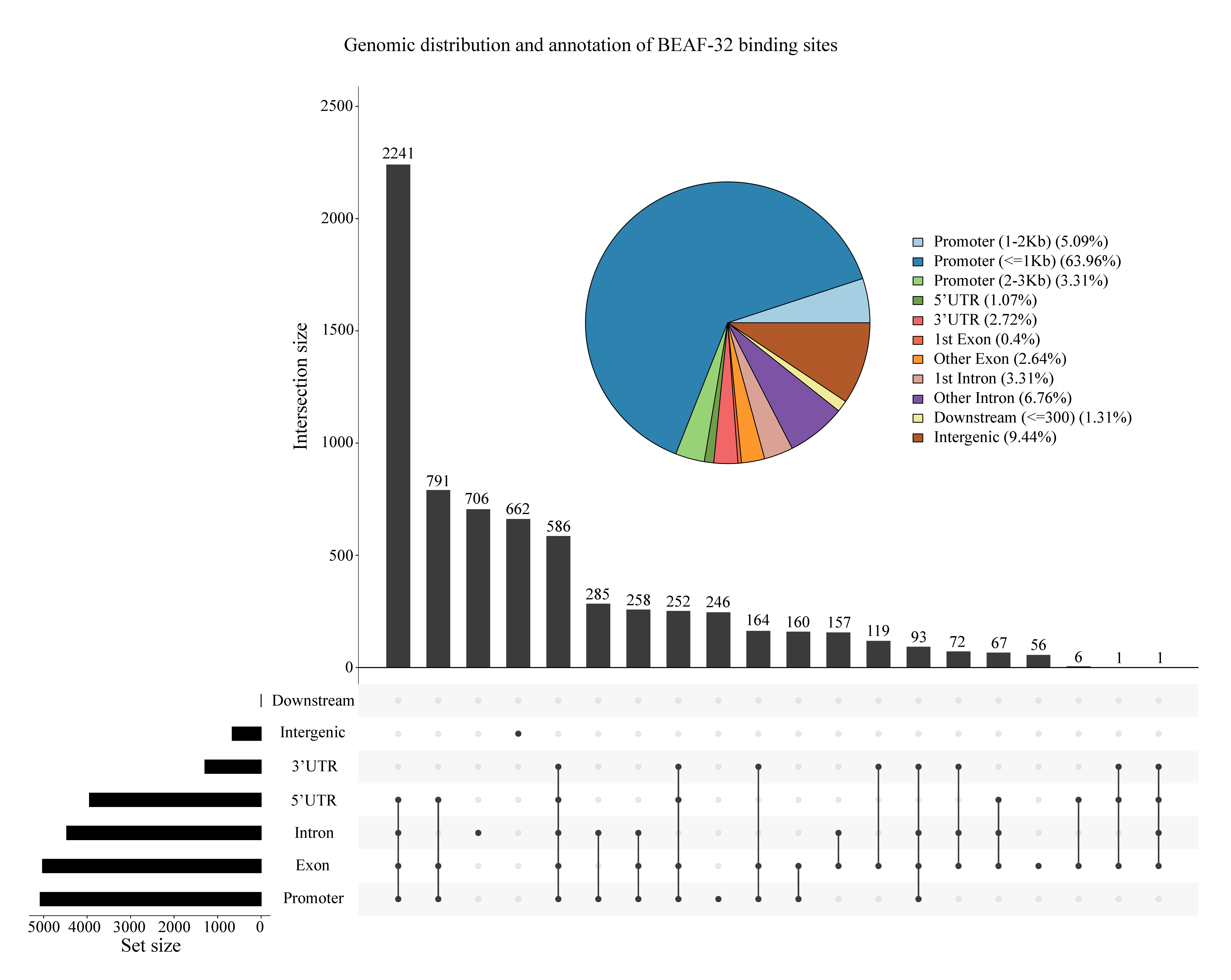

Supplement: Supplementary file 4 — Supplementary file4 (TIF 903 kb) [file 41598_2020_68879_MOESM4_ESM.tif]

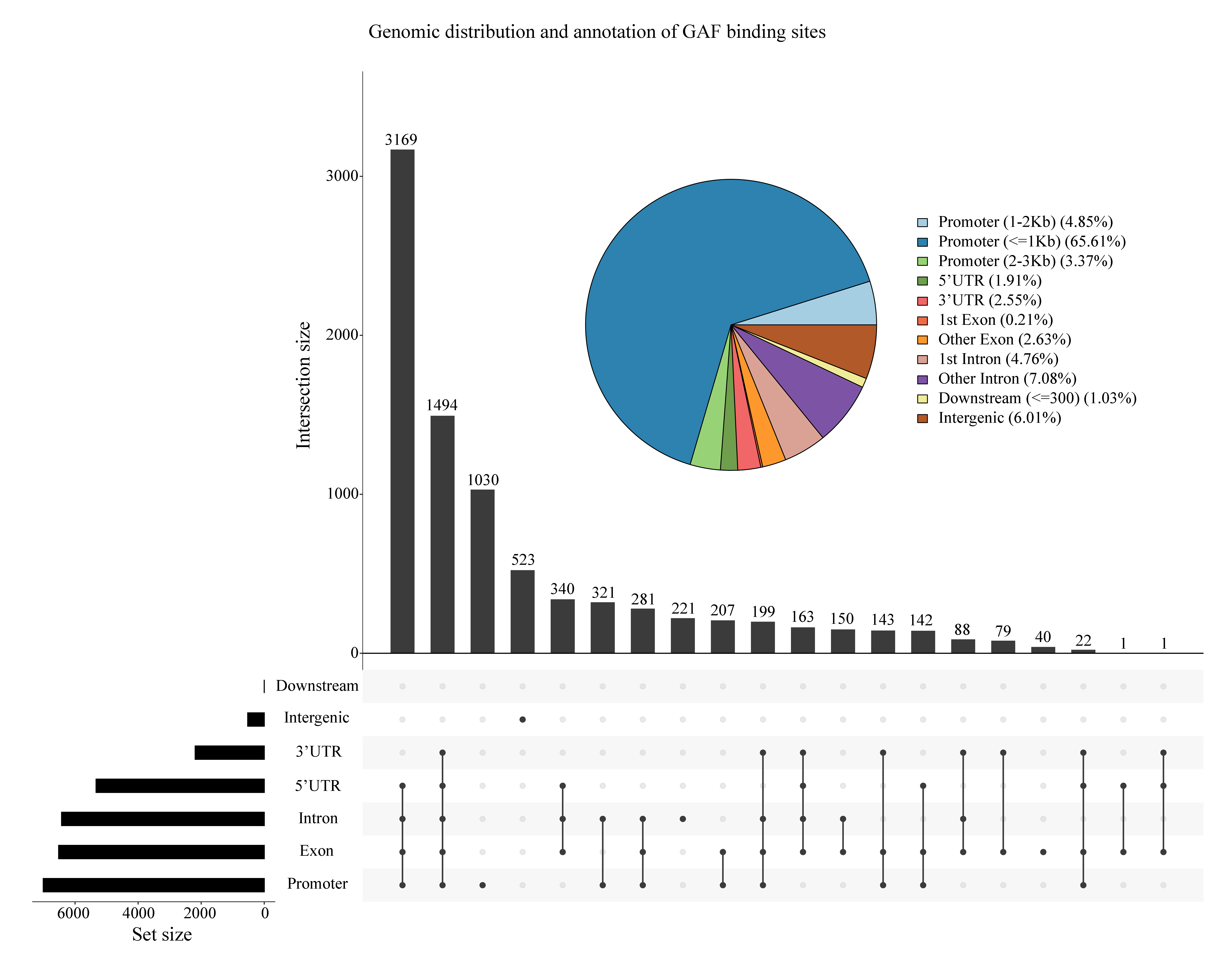

Supplement: Supplementary file 5 — Supplementary file5 (TIF 1024 kb) [file 41598_2020_68879_MOESM5_ESM.tif]

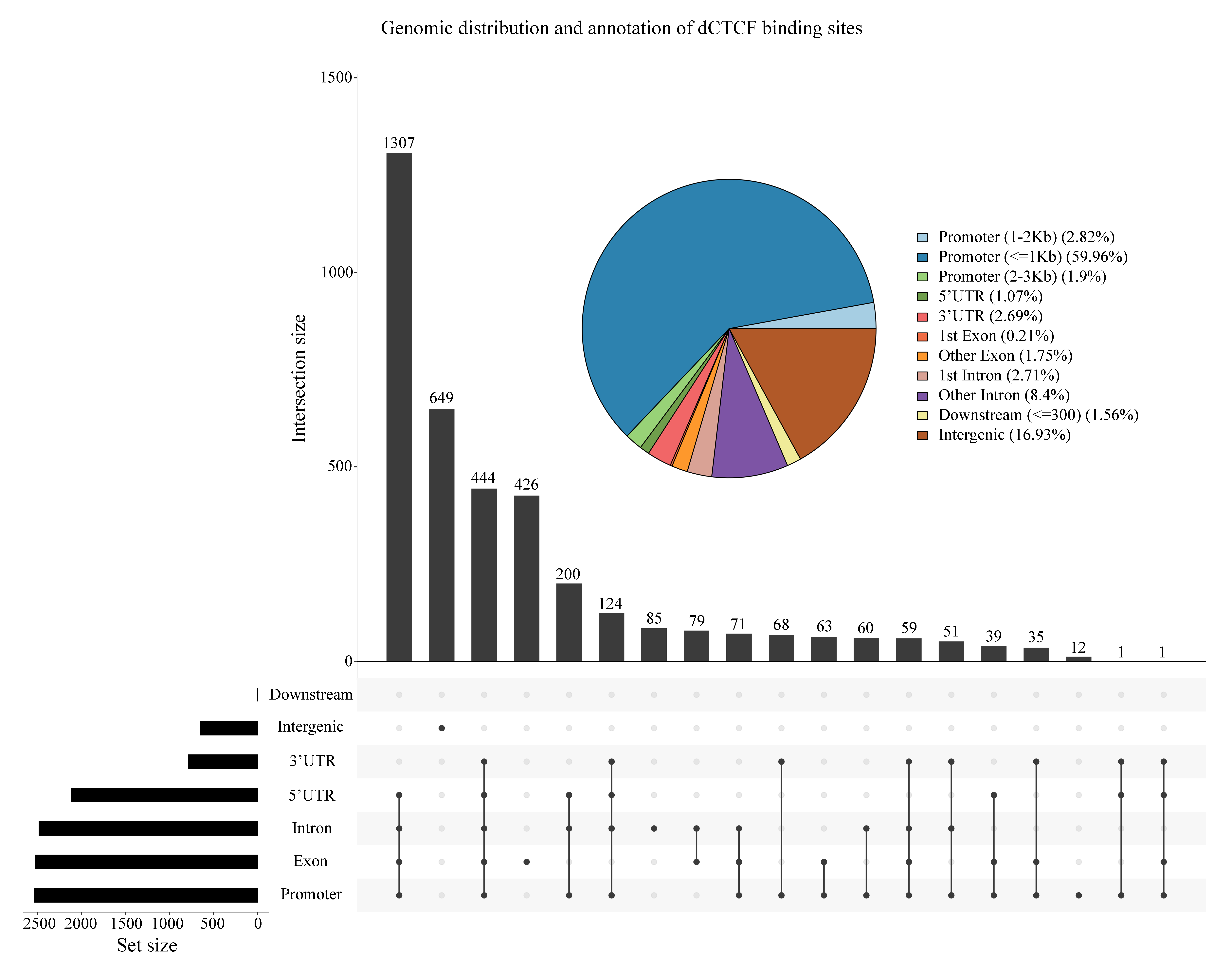

Supplement: Supplementary file 6 — Supplementary file6 (TIF 1008 kb) [file 41598_2020_68879_MOESM6_ESM.tif]

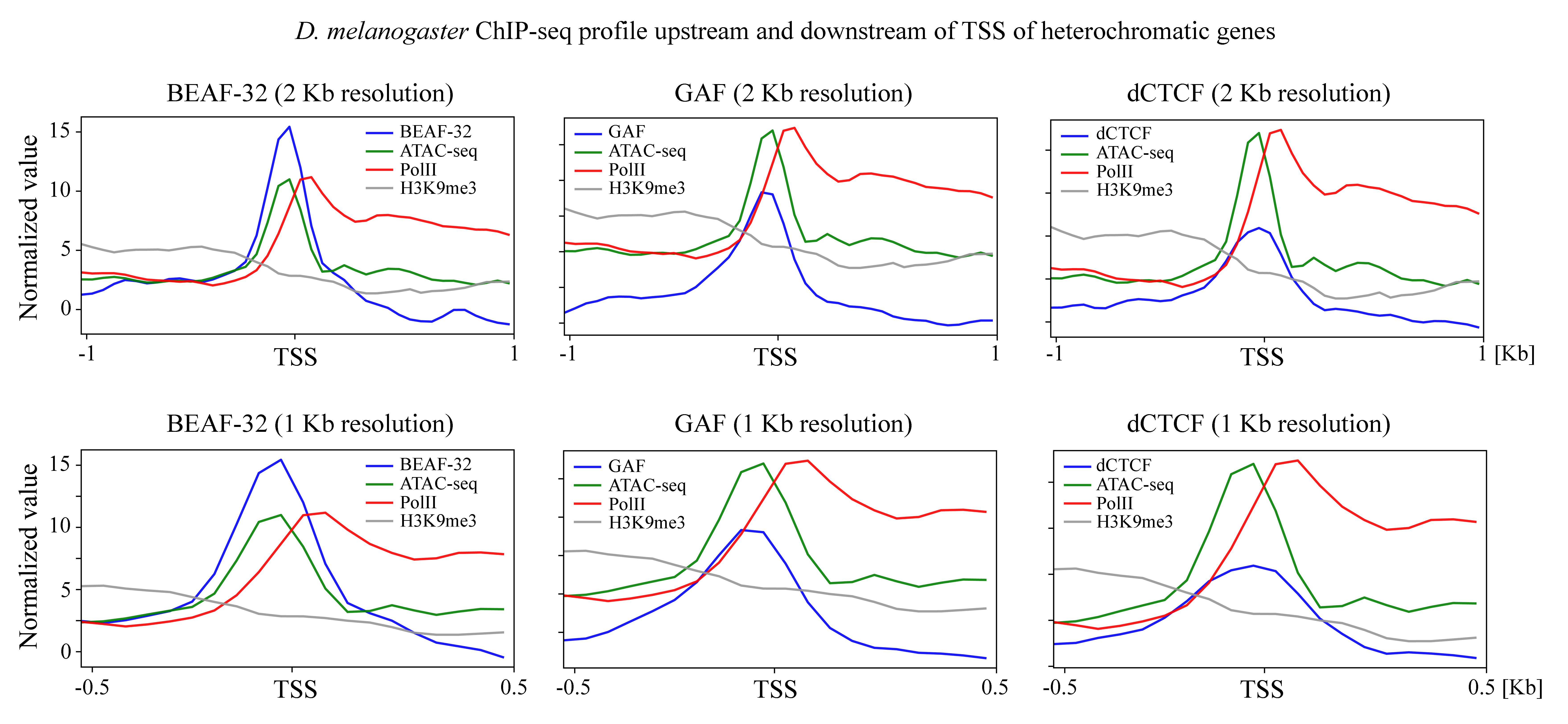

Supplement: Supplementary file 7 — Supplementary file7 (TIF 1338 kb) [file 41598_2020_68879_MOESM7_ESM.tif]

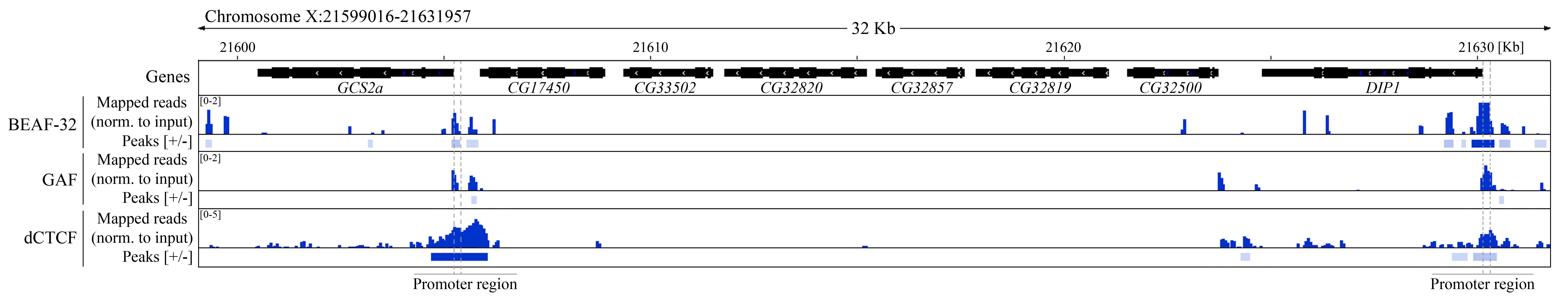

Supplement: Supplementary file 8 — Supplementary file8 (TIF 496 kb) [file 41598_2020_68879_MOESM8_ESM.tif]
