# Supplementary material for: Adaptation of gene loci to heterochromatin in the course of Drosophila evolution is associated with insulator proteins
Source: Sci Rep. 2020 Jul 17;10:11893. doi: 10.1038/s41598-020-68879-2 (PMC7368049; doi:10.1038/s41598-020-68879-2)
Supplement: Supplementary file 9 — Supplementary file9 (DOCX 17 kb) [file 41598_2020_68879_MOESM9_ESM.docx]

**Supplementary information to the manuscript entitled:**

**Adaptation of gene loci to heterochromatin in the course of *Drosophila* evolution is associated with insulator proteins.**

Sergei Yu. Funikov^1^, Alexander P. Rezvykh^1,2^, Dina A. Kulikova^3^, Elena S. Zelentsova^1^, Lyudmila A. Protsenko^1,2^, Lyubov N. Chuvakova^1^, Venera I. Tyukmaeva^4^, Irina R. Arkhipova^5^ and Michael B. Evgen’ev^1*^

^1^Engelhardt Institute of Molecular Biology of Russian Academy of Sciences, Moscow, Russia

^2^Moscow Institute of Physics and Technology, Dolgoprudny, Moscow Region, Russia

^3^Koltzov Institute of Developmental Biology of Russian Academy of Sciences, Moscow, Russia

^4^Department of Biological and Environmental Science, University of Jyväskylä, 40014, Finland

^5^Josephine Bay Paul Center for Comparative Molecular Biology and Evolution, Marine Biological Laboratory, Woods Hole, Massachusetts, USA

*Corresponding author – Michael B. Evgen’ev, Engelhardt Institute of Molecular Biology, Russian Academy of Sciences, Moscow 119991, Russia

E-mail: misha672011@yahoo.com

**Legends to supplementary figures and tables.**

Fig. S1. The content of repetitive DNA in the genomic region of *D. virilis* comprising *Myb* and *Ranbp16* genes. Classes and subclasses of transposable elements are indicated in different colors. Divergence of repeats is shown as the percentage of element coverage from the entire length of the consensus sequence of the repetitive element where 100% should be considered as full coverage or full-size element located in the considered region.

Fig. S2. The estimated evolutionary divergence (a and b) and the ratio of non-synonymous and synonymous substitutions (c and d) across Diptera species. a and b) The number of base substitutions per site (Distance) for *Myb* and *Ranbp16* genes, respectively. Standard error estimate(s) are shown above the diagonal (Standard error). Analyses were conducted using the Tamura-Nei model. All positions containing gaps were discarded. c and d) Results of dN/dS test for *Myb* and *Ranbp16* genes, respectively. Estimation was performed in relation to *D. melanogaster* sequence.

Fig. S3. The intergenic region between *Ranbp16* gene and the adjacent *Stim* gene of *D. melanogaster* exported from UCSC genome browser. CAGE-seq data indicate the beginning of 5’UTR (5’untranslated region) on each DNA strand. Top upper track shows the promoter region coordinates fetched from Eukaryotic Promoter Database (EPD) (<https://epd.epfl.ch//index.php>). Sequence conservation is shown for 27 insects presented in the UCSC database.

Fig. S4. Genomic annotation of ChIP-seq peaks of BEAF-32 in terms of genomic features in *D. melanogaster.* Pie plot indicates overall peaks annotation according to genomic features in the percentage of all observed peaks (*p*- and *q*-values < 0.05). Bar plot indicates overlaps of peaks with content of genomic features (promoter, exon, intron, 5’UTR, 3’UTR, intergenic regions).

Fig. S5. Genomic annotation of ChIP-seq peaks of GAF in terms of genomic features in *D. melanogaster.* Pie plot indicates overall peaks annotation according to genomic features in the percentage of all observed peaks (*p*- and *q*-values < 0.05). Bar plot indicates overlaps of peaks with content of genomic features (promoter, exon, intron, 5’UTR, 3’UTR, intergenic regions).

Fig. S6. Genomic annotation of ChIP-seq peaks of dCTCF in terms of genomic features in *D. melanogaster.* Pie plot indicates overall peaks annotation according to genomic features in the percentage of all observed peaks (*p*- and *q*-values < 0.05). Bar plot indicates overlaps of peaks with content of genomic features (promoter, exon, intron, 5’UTR, 3’UTR, intergenic regions).

Fig. S7. Enrichment plots of ChIP-seq reads of BEAF-32, GAF, dCTCF, RNA Pol II, H3K9me3 and ATAC-seq reads on heterochromatic genes of *D. melanogaster*. Upper panel – resolution 2 Kb around TSS, lower panel – resolution 1 Kb around TSS.

Fig. S8. Enrichment of BEAF-32, GAF and dCTCF in the heterochromatic gene cluster on chromosome X of *D. melanogaster*. ChIP-seq reads are shown in RPMs (reads per million) normalized to input samples, calculated areas of enrichment relative to the input data (peaks; *p*-value < 0.05) are shown. Promoters regions of *GCS2α* and *DIP1* are highlighted by dashed lines.

Table S1. List of the *Myb* and *Ranbp16* orthologs in Drosophila species, their coordinates in the genome and length of genes and proteins.

Table S2. List of studied *D. melanogaster* genes and their orthologs in *D. virilis* with specification of their location in the genome and chromatin type (eu- or heterochromatin).

Table S3. List of pericentric genes of D. melanogaster those promoters are occupied by BEAF-32, GAF and dCTCF in Drosophila tissue.

Table S4. List of pericentric genes of *D. melanogaster* those promoters are occupied by BEAF-32 and Dref proteins in Kc167 cells.
